# Adaptation of the *Mycobacterium tuberculosis* transcriptome to biofilm growth

**DOI:** 10.1371/journal.ppat.1012124

**Published:** 2024-04-18

**Authors:** Madison A. Youngblom, Tracy M. Smith, Holly J. Murray, Caitlin S. Pepperell

**Affiliations:** 1 Microbiology Doctoral Training Program, University of Wisconsin-Madison, Madison, Wisconsin, United States of America; 2 Department of Medical Microbiology and Immunology, School of Medicine and Public Health, University of Madison-Wisconsin, Madison, Wisconsin, United States of America; 3 Department of Medicine (Infectious Diseases), School of Medicine and Public Health, University of Wisconsin-Madison, Madison, Wisconsin, United States of America; University of Massachusetts Medical School, UNITED STATES

## Abstract

*Mycobacterium tuberculosis* (*M*. *tb*), the causative agent of tuberculosis (TB), is a leading global cause of death from infectious disease. Biofilms are increasingly recognized as a relevant growth form during *M*. *tb* infection and may impede treatment by enabling bacterial drug and immune tolerance. *M*. *tb* has a complicated regulatory network that has been well-characterized for many relevant disease states, including dormancy and hypoxia. However, despite its importance, our knowledge of the genes and pathways involved in biofilm formation is limited. Here we characterize the biofilm transcriptomes of fully virulent clinical isolates and find that the regulatory systems underlying biofilm growth vary widely between strains and are also distinct from regulatory programs associated with other environmental cues. We used experimental evolution to investigate changes to the transcriptome during adaptation to biofilm growth and found that the application of a uniform selection pressure resulted in loss of strain-to-strain variation in gene expression, resulting in a more uniform biofilm transcriptome. The adaptive trajectories of transcriptomes were shaped by the genetic background of the *M*. *tb* population leading to convergence on a sub-lineage specific transcriptome. We identified widespread upregulation of non-coding RNA (ncRNA) as a common feature of the biofilm transcriptome and hypothesize that ncRNA function in genome-wide modulation of gene expression, thereby facilitating rapid regulatory responses to new environments. These results reveal a new facet of the *M*. *tb* regulatory system and provide valuable insight into how *M*. *tb* adapts to new environments.

## Author summary

Understanding mechanisms of resistance and tolerance in *Mycobacterium tuberculosis* (*M*. *tb*) can help us develop new treatments that capitalize on *M*. *tb*’s vulnerabilities. Here we used transcriptomics to study both the regulation of biofilm formation in clinical isolates as well as how those regulatory systems adapt to new environments. We find that closely related clinical populations have diverse strategies for growth under biofilm conditions, and that genetic background plays a large role in determining the trajectory of evolution. These results have implications for future treatment strategies that may be informed by our knowledge of the evolutionary constraints on strain(s) from an individual infection. This work provides new information about the mechanisms of biofilm formation in *M*. *tb* and outlines a framework for population level approaches for studying bacterial adaptation.

## Introduction

*Mycobacterium tuberculosis* (*M*. *tb*), the causative agent of tuberculosis (TB), is a globally distributed and highly prevalent pathogen. *M*. *tb* is uniquely adapted to its environment within human tissues and is adept at evading both antibiotic treatment and the immune system, resulting in recalcitrant, difficult to treat infections. Increasing drug resistance among populations of *M*. *tb* is a global health concern: in 2021 there were over 10 million new infections, ~500,000 of which were multi-drug resistant [1]. Our ability to treat infections in the face of ever-worsening antibiotic resistance hinges on our understanding of how *M*. *tb* responds and adapts to the various selective pressures encountered during infection and transmission.

*M*. *tb*’s regulatory system is highly interconnected [2,3] and is structured such that genome-wide patterns of gene expression can be changed rapidly via master regulatory elements [2,4]. Features of this regulatory system have been well-studied under a number of conditions relevant to *M*. *tb* pathogenesis including dormancy [5] and hypoxia [6–8]. These studies have outlined regulatory programs used by *M*. *tb* to adapt to these conditions including the DosR regulon, which is responsible for coordinating gene expression during non-replicative dormancy. Non-replicative dormancy is thought to allow *M*. *tb* to persist during latent infection.

Studies of human autopsies [9–11] and animal models [12] have identified aggregated *M*. *tb* cells that resemble biofilms, suggesting that biofilms are an important growth form for *M*. *tb* during natural infection. Biofilms contribute to infection persistence via immune evasion and drug tolerance [12–15]. Yet, we know very little about biofilm formation, particularly the regulatory processes governing this growth form. A single transcriptomics study of *M*. *tb* pellicle biofilms has been published, in which the authors identified a role for isonitrile lipopeptide (INLP) in biofilm growth [16]. In a recent paper we describe adaptation of *M*. *tb* clinical isolates to biofilm growth, using an evolve and re-sequence approach. We discovered that the mutational pathway to enhanced biofilm growth varied between clinical isolates [17]. These results led us to hypothesize that the regulatory mechanisms underlying pellicle biofilm formation exhibit similar variation.

Here we use experimental evolution and high-coverage transcriptomics to delineate transcriptional responses to pellicle biofilm growth across six clinical strains belonging to two *M*. *tb* sub-lineages of lineage 4 (L4.9 and L4.2). We further quantify changes to the transcriptome after application of a uniform selective pressure for pellicle growth. We found these relatively closely related clinical strains of *M*. *tb* to have diverse transcriptional responses to biofilm growth. The application of a uniform selective pressure for pellicle growth caused the strains to converge on a shared transcriptome characterized by wide scale downregulation of gene expression. Within this global pattern we could differentiate two subtypes that were associated with bacterial sub-lineage. Strains from the same sub-lineage evolved a characteristic transcriptome, although the mutational path to this shared transcriptome varied from strain to strain. This implies that pre-existing genetic differences, even among closely related strains, have a strong impact on transcriptome adaptation. We further found that the *M*. *tb* biofilm transcriptome is characterized by widespread upregulation of non-coding RNA (ncRNA) and hypothesize that expression of ncRNA contributes to genome-wide gene expression modulation.

## Results

### RNA sequencing of clinical and experimental M. tb populations

We previously passaged six clinical populations of *M*. *tb* (isolated from sputum and not subjected to single-colony passage) under selective pressure to grow as pellicle biofilms as described in T. M. Smith et al., 2022. We identified phenotypic changes during adaptation to biofilm growth including changes in cell morphology, increased extracellular matrix (ECM) production and alterations of growth rate. We also identified genetic changes accompanying enhanced biofilm growth that varied according to the genetic background of the population [17].

**Fig 1:**
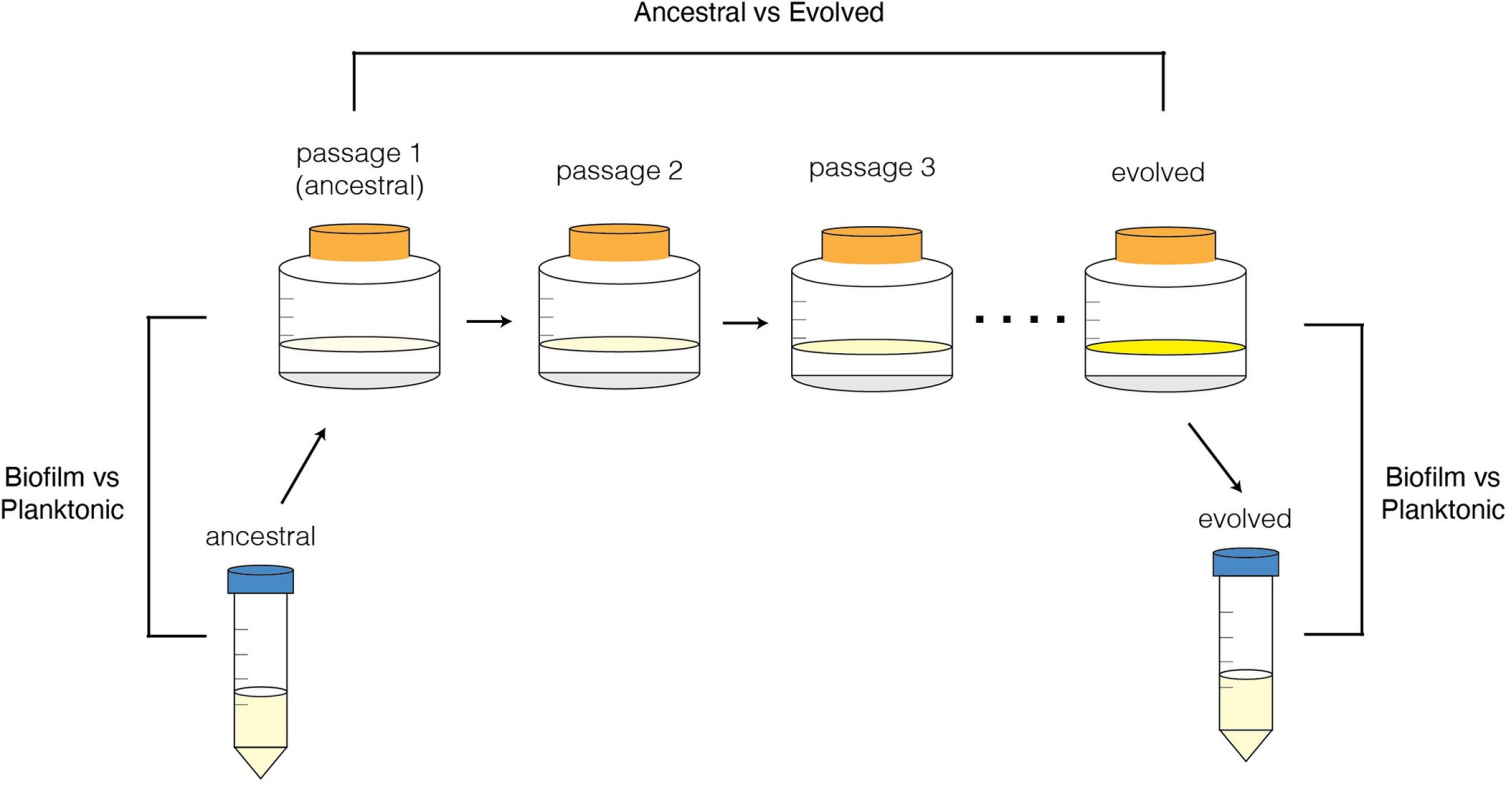
Protocol for the serial passage of *M*. *tb* pellicle biofilms and subsequent transcriptomic comparisons. Ancestral (clinical) populations were grown in planktonic cultures, then grown as pellicle biofilms (passage 1) before passaging 8–12 times. This passaged (evolved) population was taken from a biofilm culture and grown planktonically again. We performed RNA sequencing on all ancestral and evolved populations, grown under both biofilm and planktonic conditions.

Here, we sought to assess how biofilm adaptation affected the transcriptome. To investigate this question, we performed RNA sequencing on ancestral and evolved populations of *M*. *tb* from the passaging experiment, grown under biofilm and planktonic conditions (Fig 1). Two biological replicates were sequenced for each combination of population, genotype, and condition–excepting ancestral planktonic MT49, for which only one replicate was available (S1 Table). We extracted between 0.4 and 7 μg of total RNA for each sample and sequenced to high coverage: between 50–120 million reads per sample (S1 Table). Sequencing reads were quality checked, trimmed, and aligned to the *M*. *tb* H37Rv reference genome (NCBI accession NC_000962.3). For calculating expression counts we used HTSeq [18] and a custom annotation file (see Methods). Differential expression analysis was performed in R using DESeq2 [19]. No batch effects were observed (S1 Fig) in the transcriptomic data and there was overall concordance between biological replicates (S2 Fig), with two exceptions that are discussed further below.

We present two types of analyses of these RNA sequencing data: comparisons of transcriptomes from bacterial populations grown under different conditions (usually pellicle versus planktonic) and comparisons of ancestral and evolved populations grown under identical conditions (Fig 1). The type of comparison is indicated in accompanying figures with an experimental schematic.

### Diversity among M. tb biofilm transcriptomes

**Fig 2:**
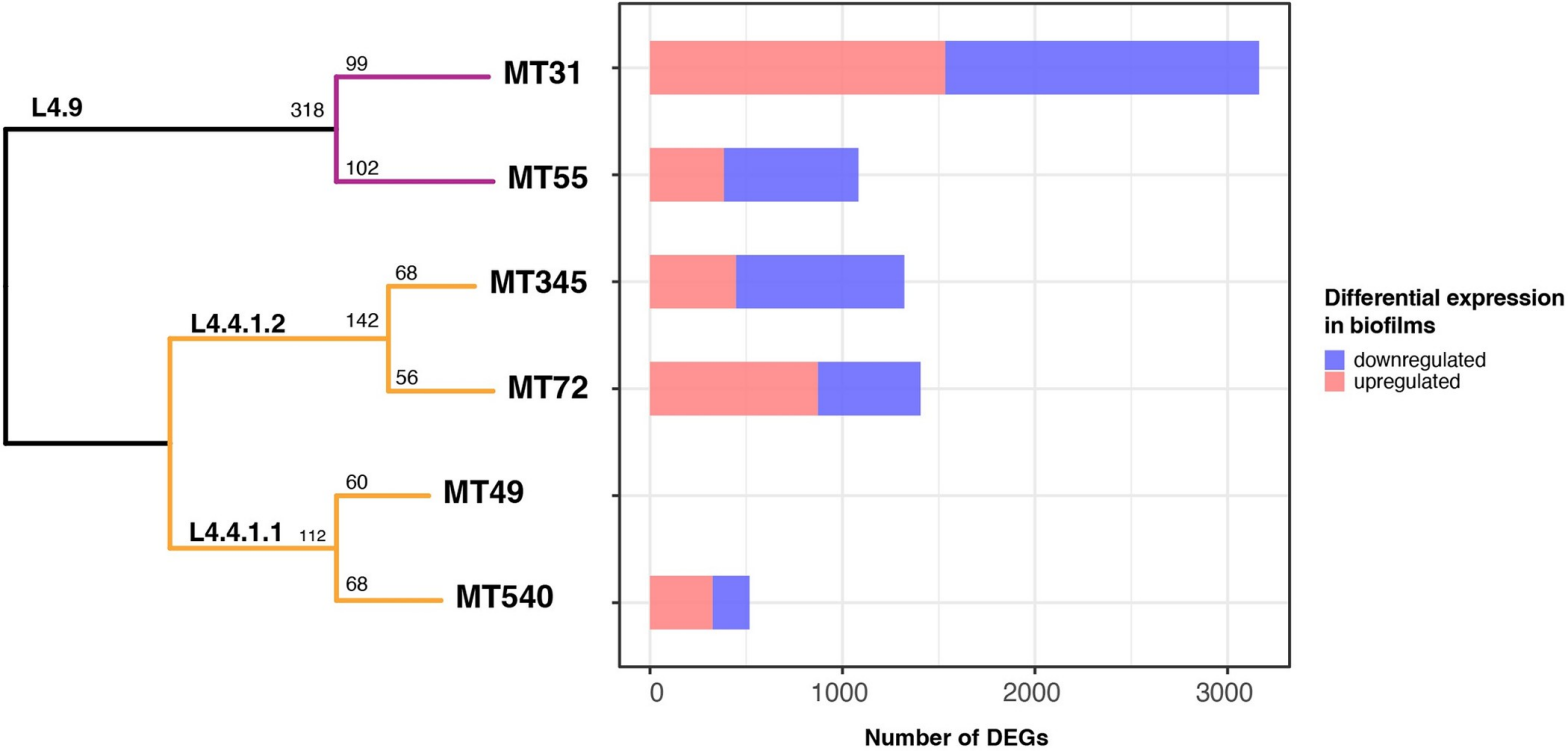
Differentiation of transcriptomes between closely related populations. Left: Whole-genome sequence phylogeny of ancestral populations used for passaging. Populations fall into two major L4 sub-lineages: L4.9 (purple) and L4.4 (orange) with SNP distances shown along each branch. Right: Number of significantly differentially expressed genes (DEGs) between biofilm and planktonic conditions for each ancestral population. Note that the analysis of ancestral MT49 is not included because only one biological replicate was available under planktonic conditions.

**Fig 3:**
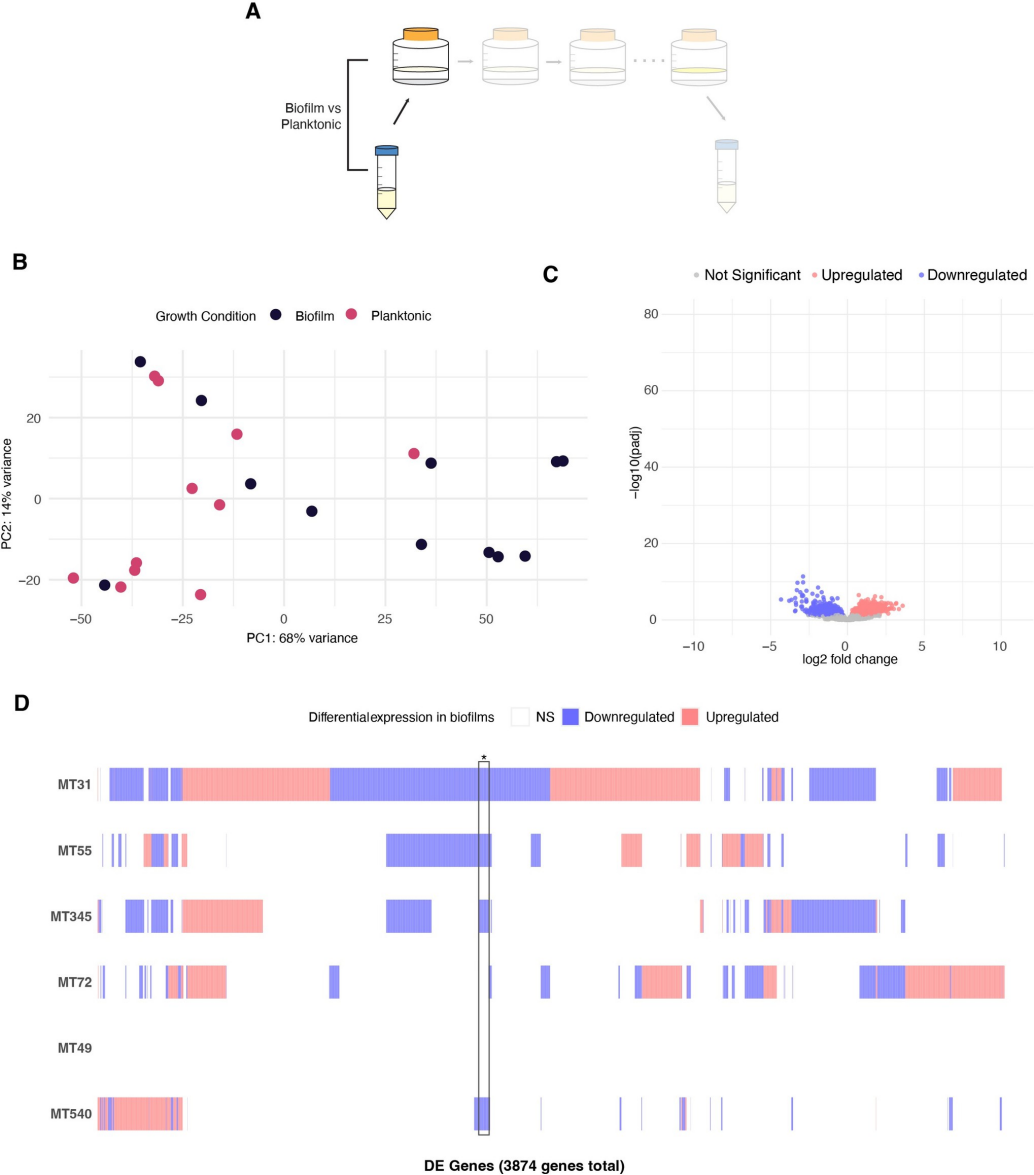
Genetic background affects transcriptional mechanisms of biofilm growth. A) Experimental diagram highlighting comparator populations: ancestral populations grown as pellicle biofilms are compared to the same populations grown in planktonic culture. Ancestral populations from freezer stocks of low-passage clinical isolates were used to seed planktonic cultures, and subsequently biofilm cultures. B) Principal component analysis (PCA) of variance stabilizing transformed gene expression for ancestral populations. Each point is a biological replicate. Results demonstrate diversity among ancestral transcriptomes, with intermingling of planktonic and biofilm samples. C) Volcano plot summarizing differential expression between ancestral populations grown as biofilms and as planktonic cultures. Log transformed adjusted p-values plotted against the log2 fold change for each gene. Genes that did not have significant differential expression are shown in grey. Very few genes are significantly differentially expressed when all populations are analyzed together. A zoomed in version of this figure is available in S4D Fig) Matrix of individual DEGs shared across ancestral populations. A total of 3874 DEGs are plotted according to whether the gene is upregulated (red), downregulated (blue) or not significantly differentially expressed (white) in each population. *Only 4% of downregulated genes are shared by at least 4 of the analyzed populations while no upregulated genes are shared across more than 3 populations. Note that analyses of ancestral MT49 are not included as only one biological replicate was available under planktonic conditions.

We compared the transcriptomes of ancestral bacterial populations grown under biofilm and planktonic conditions. Genetic background appears to have a substantial impact on these transcriptomes, which were consistent among biological replicates but differentiated between populations (Fig 2), with intermingling of planktonic and biofilm samples (Figs 3B and S3A). When analyzed individually, our ancestral populations had a total of ~ 500–3000 DEGs separating biofilm and planktonic conditions (Fig 2 and S1 Data). In looking at DEGs with more extreme values (log2 fold change more than 2), we detected only 143 DEGs when all populations were analyzed together (Figs 3C and S4), versus an average of 625 genes when the populations were analyzed separately (S1 Data). There is very little overlap in DEGs between populations: only 4% of downregulated genes are shared by at least five populations and no upregulated genes are shared across more than four populations (Fig 3D). To assess whether the effect of genetic background was reflective of phenotypic differences between ancestral populations, we overlayed wet weight measurements (the simplest marker of biofilm phenotype, which measures the total biomass of the biofilm) onto normalized, log-transformed gene expression (S3B Fig). We did not identify a simple relationship between transcriptomic signature and biofilm mass, as samples with similar biomass were spread across transcriptomic space summarized in the PCA. Overall, our results indicate that biofilm transcriptomes vary among closely related strains of *M*. *tb*, separated by just 10s-100s of SNPs across the genome (Fig 2).

### Biofilm passaging decreases transcriptome diversity and intensifies repression of gene expression

**Fig 4:**
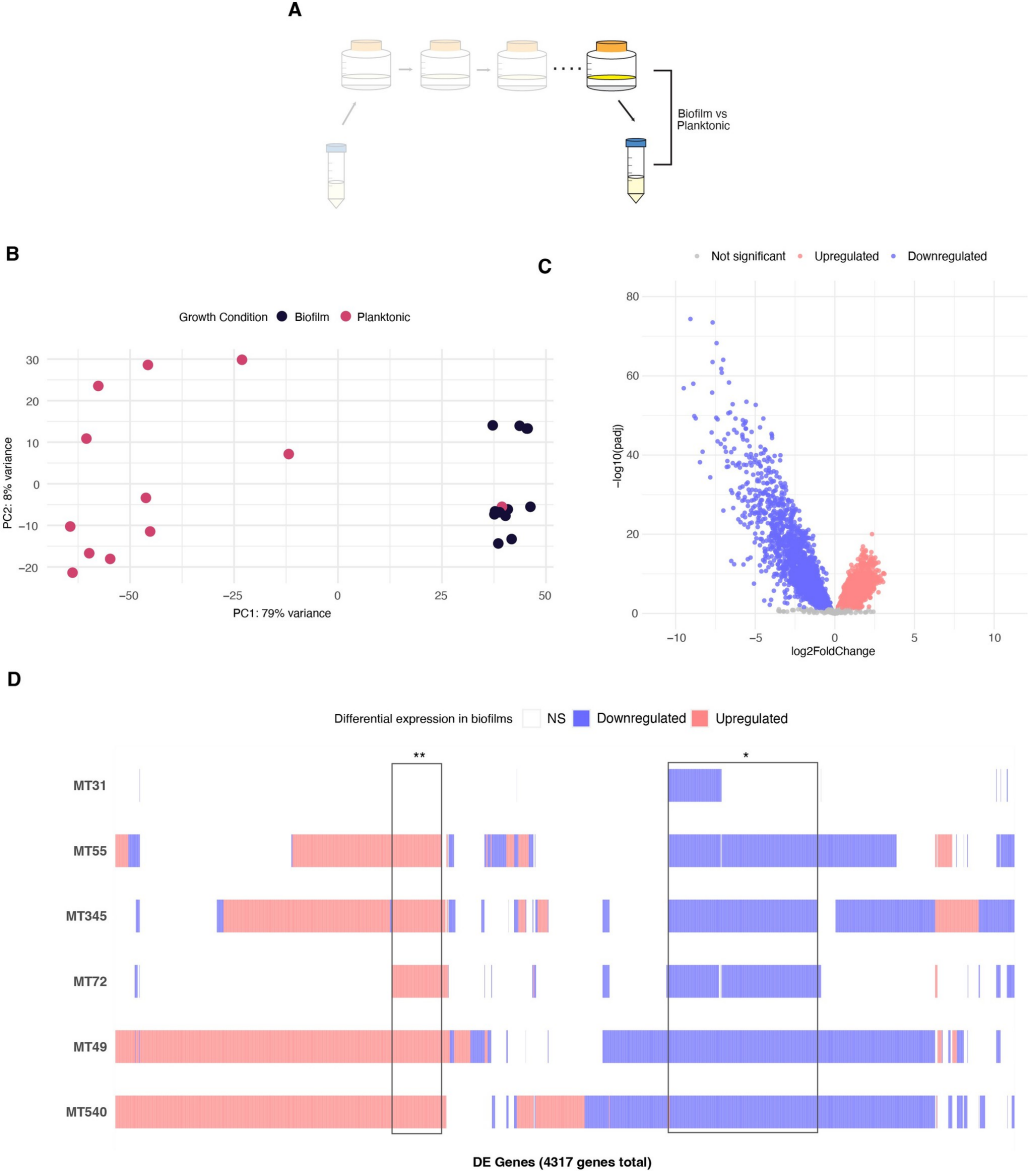
Transcriptional responses to biofilm growth following biofilm passaging. A) Experimental diagram highlighting comparator populations: evolved populations grown as pellicle biofilms are compared to the same populations grown in planktonic cultures. Analyses are parallel to those of ancestral populations in Fig 3. B) Principal component analysis (PCA) of variance stabilizing transformed gene expression for evolved populations grown as biofilms and planktonic cultures. Biofilm passaging resulted in clear separation of biofilm and planktonic transcriptomes. These transcriptomes were not clearly separated at baseline (Fig 3B) C) Volcano plot summarizing differential expression between evolved populations grown as biofilms and as planktonic cultures. Log transformed adjusted p-values plotted against the log2 fold change for each gene. Genes that did not have significant differential expression are shown in grey. Biofilm transcriptomes were characterized by broad-scale downregulation of gene expression. D) Matrix of individual DEGs shared across evolved populations. A total of 3568 DEGs are plotted according to whether that gene is upregulated (red), downregulated (blue) or not significantly differentially expressed (white) in each population. 17% of downregulated genes are shared by at least 5 populations (*), while 6% of upregulated genes are shared by at least 5 populations (**); see S2 Data for details. This contrasts with ancestral populations, which did not share any upregulated genes and only shared 4% of downregulated genes across 5 or more populations (Fig 3D). Note that low concordance between biological replicates of evolved MT31 grown under planktonic conditions may affect these results. See text for further discussion.

Prior to biofilm passaging, we observed substantial diversity among biofilm transcriptomes in our sample (Figs 2 and 3). The application of a uniform selection pressure resulted in a significantly more uniform biofilm transcriptome across genetic backgrounds (S5 Fig), which was clearly distinguishable from planktonic transcriptomes: approximately 76% of variance in our samples can be attributed to growth condition (represented by PC1, Fig 4B). Transcriptome uniformity was specific to biofilm growth, as inter-sample distance actually increased after passaging when bacteria were grown in planktonic culture (S5 Fig). When all populations were analyzed together, approximately 75% of the genome was involved in the transcriptomic response to biofilm conditions, with ~ 3600 genes differentially expressed in comparison with planktonic growth (Fig 4C and S2 Data). In comparison with ancestral populations, the evolved biofilm transcriptome was characterized by more extensive repression of gene expression (Figs 3C and 4C). Looking at the expression of individual genes, gene expression in evolved populations appears more homogenous than in ancestral populations (S6 Fig). This greater uniformity is also reflected in a larger proportion of shared, downregulated DEGs across populations: 17% in the evolved versus 4% in the ancestral populations (Figs 3D and 4D). We also observed a small proportion (6%) of DEGs that were upregulated across five populations, suggesting uniform recruitment of a cadre of genes under selection pressures imposed in our experiment (Fig 4D). DEGs that are shared across five or more populations are shown in S2 Data. Comparison with published surveys of candidate *M*. *tb* biofilm loci indicates that the vast majority (97%) of candidate loci identified in the present study are novel (S2 Data).

Two biological replicates are outliers with respect to their inter-replicate distance (S2 Fig): ancestral MT72 grown as a biofilm (S3A Fig) and evolved MT31 grown planktonically (S5A Fig). In the case of evolved MT31, it is clear that one of the replicates has a gene expression profile more similar to a biofilm than to other planktonic samples (Figs 4B and S6B). If we classify this sample as planktonic, we identify few DEGs within this population (Fig 4D). If we instead group planktonic MT31 with the biofilm samples, we see a stronger contrast between conditions, and a much higher number of DEGs for MT31, many of which are shared with the other biofilm populations (S7 Fig). After extended passaging as a biofilm, we attempted to grow our populations planktonically again and while most populations retained the capacity to grow shift out of a biofilm-like transcriptome, this replicate did not. Interestingly, this population was not defective for growth under planktonic conditions (as measured by OD600) and in fact evolved a higher rate of planktonic growth in response to biofilm passaging [17]. In our experience with *M*. *tb* clinical strains, we have observed that they grow less reproducibly than H37Rv, which likely reflects their limited exposure to laboratory conditions compared with the lab-adapted reference strain. Unlike H37Rv, strong biofilm formers often develop biofilm-like clumps in planktonic culture, even in the presence of detergents and shaking. Clumping may explain why this particular replicate had a biofilm-like transcriptome, despite being grown in planktonic culture. Clumping also introduces variability in measurements of growth, which could affect sample to sample variability. Conversely, poor biofilm formers (like ancestral MT72) may retain the transcriptional signatures of planktonic culture even under biofilm growth conditions. We hypothesize that the variability of transcriptomic data from these two samples reflects generally inconsistent in vitro growth habits of clinical strains (and their derivatives).

### Pellicle transcriptomes are distinct from stationary phase and minimal media transcriptomes

Pellicle and planktonic conditions differ with respect to both media and duration of culture. Thus, transcriptional differences between these conditions may reflect the impacts of nutritional milieu and growth phase, in addition to dispersed versus biofilm growth. We used the relatively tractable laboratory reference strain H37Rv to dissect the influences of media, growth phase and state on *M*. *tb* transcriptomes. We compared transcriptomic data from H37Rv grown under the following conditions: standard planktonic, stationary phase in 7H9 with detergent and shaking, Sauton’s media with detergent and shaking, and standard pellicle. Data from biological replicates within each condition were tightly correlated (S8A Fig). The minimal media (Sauton’s) transcriptome was characterized by more widespread repression of gene expression relative to pellicle and stationary phase growth (S8B Fig). PCA summary of transcriptomic data indicates that the planktonic/exponential phase, stationary phase, minimal media and pellicle transcriptomes are clearly distinct (S8 Fig and S3 Data).

Using genes implicated in both biofilm and minimal media/stationary phase growth, we looked for commonalities with the “evolved pellicle transcriptome” (DEGs shared by five or more evolved populations). Of 952 genes in the evolved pellicle transcriptome, only 47 are common to the biofilm and minimal media/stationary phase transcriptomes in H37Rv (S2 Data). These commonalities include nine genes that are upregulated in both the evolved pellicle transcriptome and stationary phase culture including two ncRNA (*ncRv0918c*, *ncRv3543*) and the transcriptional regulator *whiB7* (S2 Data).

To further investigate this phenomenon in our study isolates, we grew evolved populations to stationary phase in 7H9 with detergent and shaking, as well in Sauton’s with detergent and shaking. We were able to obtain a dispersed culture of MT31 under stationary phase conditions; otherwise this and other tested strains failed to form suspensions when grown in Sauton’s and/or 7H9 under stationary phase conditions. This likely reflects the strong orientation of these strains toward biofilm formation under a variety of conditions. We compared transcriptomic data from MT31 grown to stationary phase with exponential phase data and then looked for overlap between these data and comparisons of pellicle and planktonic transcriptomes for this strain (the RNA sequencing data from these two experiments were generated with different library preparation and sequencing technologies and thus could not be directly compared). In MT31, there was no overlap between DEGs identified in these two analyses (S8B Fig and S3 Data), supporting our results from H37Rv that the biofilm transcriptome is distinct from both stationary phase and minimal media transcriptomes.

### Genetic background shapes adaptive trajectories

**Fig 5:**
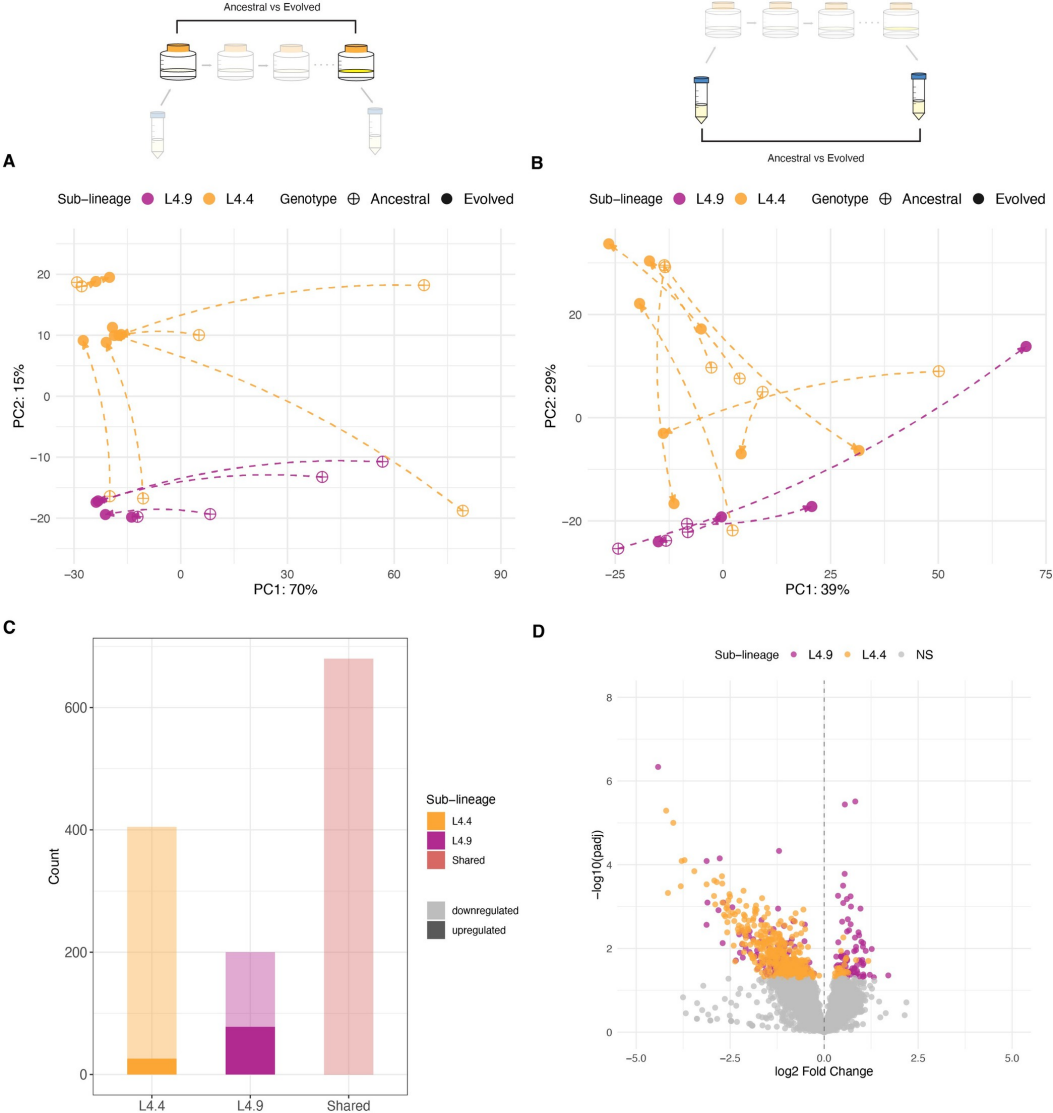
Genetic background shapes adaptive trajectories. A) Principal component analysis (PCA) of variance stabilizing transformed expression counts from ancestral and evolved biofilm populations. Arrows are drawn between corresponding ancestral and evolved populations, indicating the trajectory of evolution across passaging. Points are colored by sub-lineage of the ancestral population. B) PCA of variance stabilizing transformed expression counts from ancestral and evolved populations grown under planktonic conditions. Like panel A, arrows are drawn between corresponding ancestral and evolved populations and are colored by sub-lineage. Biofilm passaging reduced transcriptome diversity, with populations converging on a sub-lineage-specific signature (A); this pattern was not observed under planktonic growth conditions (B). N.B. While the positions of ancestral and evolved states are known (dots on the PCA), the trajectories between them are not, and connecting lines shown here are schematic. C) Bar plot of DEGs comparing evolved and ancestral biofilm populations broken down by sub-lineage. There are 680 DEGs shared between sub-lineages, and 405 and 200 DEGs unique to L4.4 and L4.9, respectively. Opacity of the bar indicates the subset of DEGs that are either up (dark) or downregulated (light) after passaging. The pellicle biofilm transcriptome is characterized by broad scale downregulation of gene expression with a smaller complement of genes that are upregulated in a sub-lineage specific manner. D) Differential expression of DEGs unique to each sub-lineage, from the comparison of evolved to ancestral biofilm populations. Log transformed adjusted p-values plotted against the log2 fold change for each gene. Genes that did not have significant differential expression are shown in grey. Lineage 4.9 evolved a larger complement of upregulated genes in response to biofilm passaging.

To further investigate how patterns of biofilm gene expression changed after passaging, we compared transcriptomes of *M*. *tb* passaged under selection for biofilm growth with their corresponding ancestral populations (Fig 5). These comparisons are concordant with respect to growth conditions, i.e. pellicle populations are compared with pellicle and planktonic with planktonic. Comparison of ancestral with evolved populations revealed three striking patterns. First, passaging under selection for biofilm growth reduced overall transcriptome diversity as summarized in a PCA (Fig 5A). This parallels the contraction of diversity observed when evolved populations were compared in pellicle versus planktonic growth conditions (Figs 4 and S5). Second, convergence of transcriptomes occurred in a lineage-specific pattern, with the evolution of clearly differentiated groups that corresponded with sub-lineage designations (Fig 5A). Finally, we found this pattern to be specific to biofilm growth as the transcriptomes of evolved populations grown planktonically remained intermingled and diverse (Fig 5B). These results indicate that transcriptomic responses were specific to the selection pressure applied, and that adaptation to this new environment was constrained by bacterial genetic background.

Gene-by-gene analyses of the transcriptome also revealed lineage-specific patterns. As noted above, biofilm growth was associated with widespread downregulation of gene expression (compared to exponential growth in planktonic culture). Biofilm passaging reinforced this pattern, with comparisons of evolved and ancestral populations revealing further genome-wide downregulation (Fig 5C and 5D). All differentially expressed genes (DEGs) that were shared among sub-lineages were downregulated (Fig 5C). Outside of these shared DEGs, the sub-lineages exhibited distinct patterns with sub-lineage 4.9 showing a larger proportion (39%) of upregulated genes in its DEG repertoire than sub-lineage 4.4 (6%) (Fig 5C and 5D and S4 Data). We found previously that *M*. *tb* populations within sub-lineage 4.9 all evolved large genomic duplications [17], raising the possibility that the relatively large proportion of upregulated genes observed here arose from the duplication. However, the majority (55%) of L4.9-specific, upregulated DEGs lie outside the duplicated region (S4 Data). Thus, the increased proportion of upregulated genes in L4.9 does not appear to arise directly from increased gene dosage from the genomic duplication. It is also noteworthy that while all populations in L4.4 evolved similar transcriptomes (Fig 5A), we did not identify any genomic mutations that were shared across these populations [17]. Together, these observations implicate a complex relationship between genetic backgrounds, novel mutations, transcriptional responses, and phenotypes. It appears that the application of a uniform selection pressure can result in similar phenotypic adaptation (in this case enhanced biofilm growth) via distinct mutational paths, that result in similar transcriptional responses. Thus, when exposed to a new environment, sub-populations of *M*. *tb* may adapt similarly using different paths to the same goal.

### Impacts of biofilm associated mutations on gene expression

In our passaging experiment, a near identical genomic duplication emerged repeatedly and specifically in association with biofilm passage, providing powerful evidence for its association with biofilm growth [17]. We have termed this the ‘MMMC duplication’ (Roman numeral 3100), an umbrella term for duplications starting around 3.1Mb (*Rv3100)* with lengths from 150–350 kb, which have been described before both *in vitro* [20–23], and *in vivo* [24]. In our experiments, the independently arising MMMC duplications became fixed in biofilm passaged populations and were stable for up to 20 passages, whereas they were absent from populations passaged as planktonic cultures [17]. Our observation that duplications in this region of the *M*. *tb* genome appear repeatedly in association with a selective pressure to grow as a biofilm suggest that dosage of genes and elements affected by the duplication are important for robust biofilm formation. Here we sought to investigate the impact of this large-scale duplication on the biofilm transcriptome, thereby illuminating potential mechanisms for its impact on biofilm growth.

**Fig 6:**
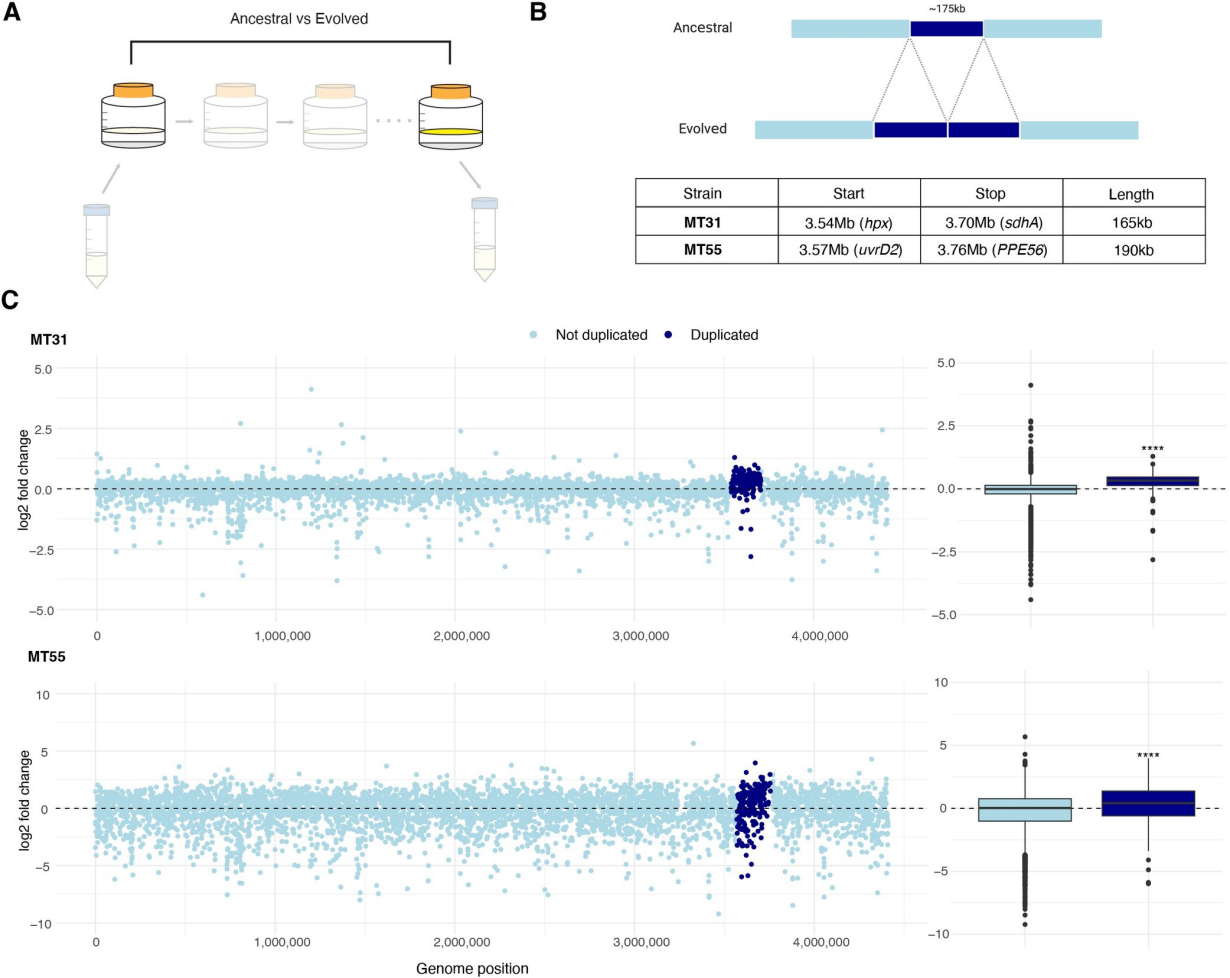
Genome duplication results in complex patterns of gene expression. A) Experimental diagram highlighting comparator populations: evolved and ancestral biofilm populations. B) Top: large tandem duplication that evolved in populations MT31 and MT55 under selection to grow as a biofilm. Bottom: coordinates of the duplication vary slightly between populations, with an average duplicated length of 175 kb. C) Left: log2 fold changes in gene expression between evolved and ancestral biofilm populations plotted against the position in the genome. Each point is a single gene, colored according to whether that gene lies inside of the duplicated region for that population. Right: Boxplots of log2 fold changes in gene expression, for genes outside (light blue) and inside (dark blue) the duplication. Genes within the duplication were significantly more likely to be upregulated in both populations (Mann Whitney U test with Benjamini-Hochberg correction, *p* < 0.0001).

A more in-depth analysis of the coordinates of the MMMC duplications using sliding-window DNA sequencing coverage revealed them to be larger than we had originally reported (~175 versus 120 kb) using another method of structural variant identification [17]. The duplication has slightly different coordinates in MT 31 and MT55, but spans from position ~3.54 to 3.76 Mb, with an average length of 175 kb, and includes genes *hpx* through *PPE56* (Fig 6B). Analyses of the transcriptomic data revealed what is likely a transient duplication in an additional population, MT49, that arose during non-specific handling of the culture, in addition to the biofilm-associated duplications in MT31 and MT55 (See *S1 Text* for details). A priori, we might expect that a second copy of a given gene would increase expression by 2x, corresponding to a log2 fold change (L2FC) value of 1. Such a modest fold change is not likely to be identified as statistically significant, and in fact only 39 duplicated genes were significantly upregulated in either MT31 or MT55 (S4 Data). To further investigate impacts of the MMMC duplication on gene expression, we plotted L2FC values between evolved and ancestral biofilm populations (Fig 6A) for all genes across the genome and compared changes in duplicated genes to non-duplicated genes (Fig 6C). Overall patterns of L2FC values seem to differ between the two populations: MT55 has greater differentiation between ancestral and evolved populations, resulting in L2FC values of higher magnitude than MT31 (Fig 6C). The distribution of L2FC values within the duplication is shifted upward relative to genes outside the duplication, a difference that was statistically significant under biofilm and planktonic growth conditions (Mann Whitney U test with Benjamini-Hochberg correction, *p* < 0.0001) for both MT31 and MT55 (Figs 6C and S9B). However, we did not observe a simple two-fold increase in gene expression within the duplication: genes exhibited a range of values with some strongly down- and up-regulated.

Another potential mechanism for the duplication’s impact on the transcriptome is via changes in the dosage of transcription factors (TFs) relative to their binding sites (TFBS). We used data from published TF overexpression studies [25] to test this hypothesis. Similar to changes in gene dosage, the data did not reveal any simple relationships between observed transcriptomic changes and TFs and TFBS within the duplication. Expression of nine out of ten TFs within the duplication’s borders was either unchanged (n = 6) or downregulated (n = 3) following their duplication. The one modestly upregulated TF, *sigF*, did not affect downstream gene expression as predicted in TFOE studies. We also examined TFs encoded outside the duplication that had a large number (> = 10) of TFBS within the duplication’s borders, which could be subject to dilution as a result of changes in the ratio of TF to TFBS following duplication. We did not observe effects consistent with TF dilution (i.e. downregulation) on downstream gene expression: of 712 genes potentially subject to TF dilution, expression of a small minority (<1%) was in the direction predicted, whereas expression of 19 was in the opposite direction predicted, and 686 genes were unaffected. We conclude that the transcriptomic impacts of the tandem duplication are complex, and hypothesize that they arise from emergent properties of *M*. *tb*’s regulatory network. In our original study we also identified biofilm-associated, intergenic SNPs at a TFBS: as with the tandem duplications, analyses of transcriptomic data revealed complex impacts of these mutations on gene expression (*S1 Text*).

### Non-coding RNA and small proteins are part of the adaptive transcriptome

Advances in transcriptomic and proteomic methods have revealed an abundance of non-coding RNA (ncRNA) [26–32] and small open reading frames (sORFs) ([33,34] in the *M*. *tb* genome. In *M*. *tb*, ncRNA are important for gene regulation and adaptation [35] and virulence [36]. In other bacteria, diversity in the non-coding transcriptome is hypothesized to be a driver of inter-strain divergence and adaptation [37]. We hypothesized that these features may similarly play a key role in *M*. *tb* adaptation to biofilm growth, and perhaps provide a mechanistic link between biofilm-associated mutations and adaptation of the transcriptome. To investigate further, we included a custom list of ncRNA and sORFs (S5 Data) in our annotation file used for all differential expression analyses in Figs 2–6. In order to identify subtle patterns in expression of these non-canonical features we separated them from the rest of the open reading frames (ORFs) and quantified their expression across conditions in the study.

**Fig 7:**
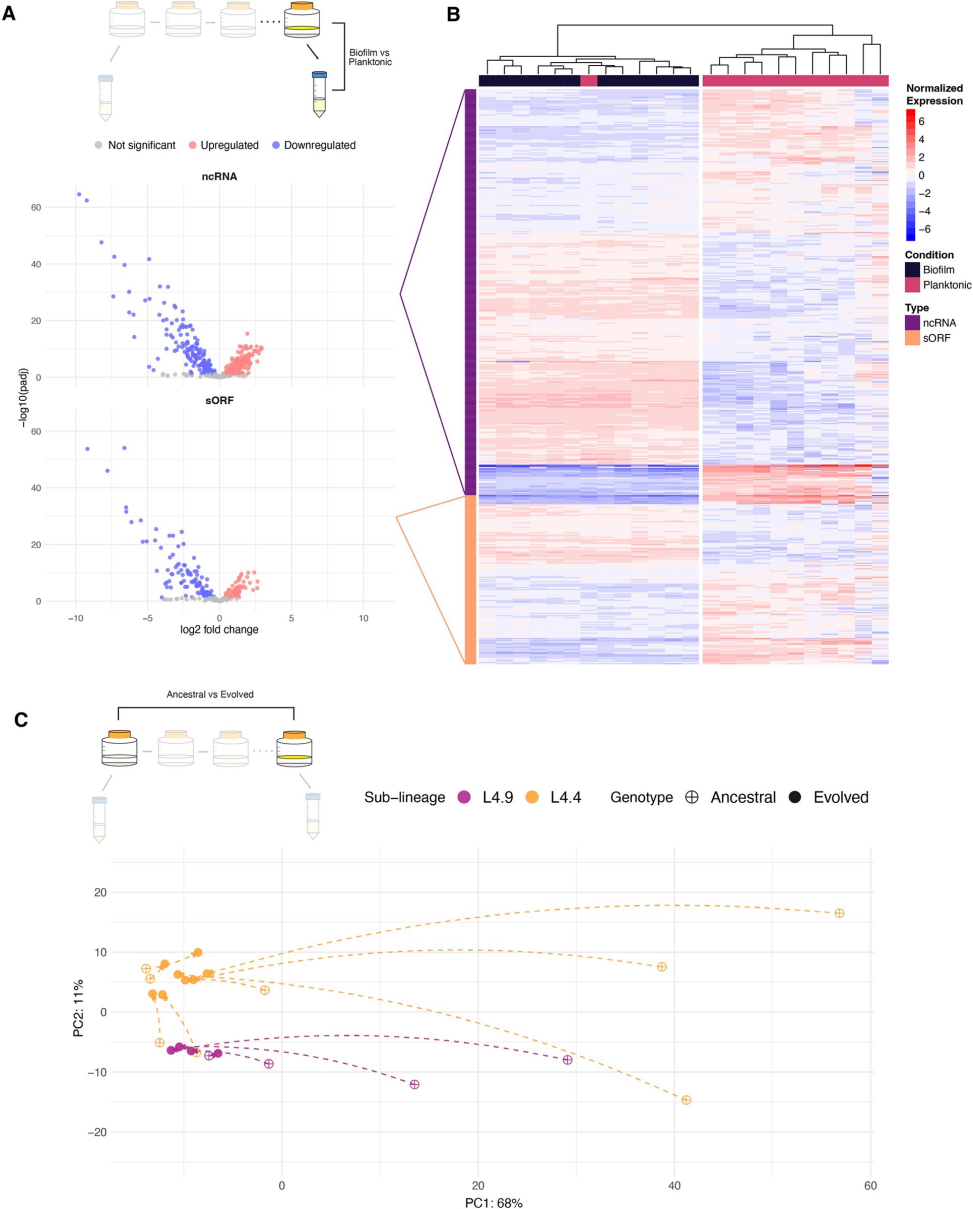
Expression of ncRNAs and sORFs mirror coding regions after passaging. A) Volcano plot summarizing differential expression of ncRNAs and sORFs in evolved populations. Log transformed adjusted p-values plotted against the log2 fold change for each gene. Genes that did not have significant differential expression are shown in grey. B) Heatmap of normalized, variance stabilizing transformed expression counts for ncRNAs and sORFs with significant differential expression in evolved populations. Each column is a single sample from an evolved population, grown either as a biofilm or in a planktonic culture. Each row is a gene labeled either ncRNA or sORF. Expression values for each gene are normalized to the mean across samples. Samples are clustered by Euclidean distance and plotted as a tree at the top of the heatmap. C) Principal component analysis (PCA) of variance stabilizing transformed expression counts of ncRNAs and sORFs from ancestral and evolved biofilm populations. Arrows are drawn between corresponding ancestral and evolved populations, indicating the trajectory of evolution across passaging. Points are colored by sub-lineage of the ancestral population. The effects of biofilm passage on ncRNAs and sORFs mirror that of coding regions: reduced transcriptome diversity and widespread downregulation with populations converging on a sub-lineage-specific signature. N.B. While the positions of ancestral and evolved states are known (dots on the PCA), the trajectories between them are not, and connecting lines shown here are schematic.

We observed that in general, sORFs and ncRNA follow similar patterns to coding regions. In comparisons of pellicle with planktonic growth, there is more downregulation (both in the number of the features and the magnitude of differential expression) than upregulation (Fig 7A and 7B). Across passaging the expression of sORFs and ncRNA converge in a genetic background dependent manner (Fig 7C), a phenomenon that does not hold true under planktonic growth conditions (S7 Fig). This mirrors what we see with transcriptome-wide adaptation to biofilm growth, which is that specific, directional changes in gene expression resulting from an applied selective pressure (biofilm growth in this case), are only observed when that selective pressure is maintained (Fig 5A and 5B).

**Fig 8:**
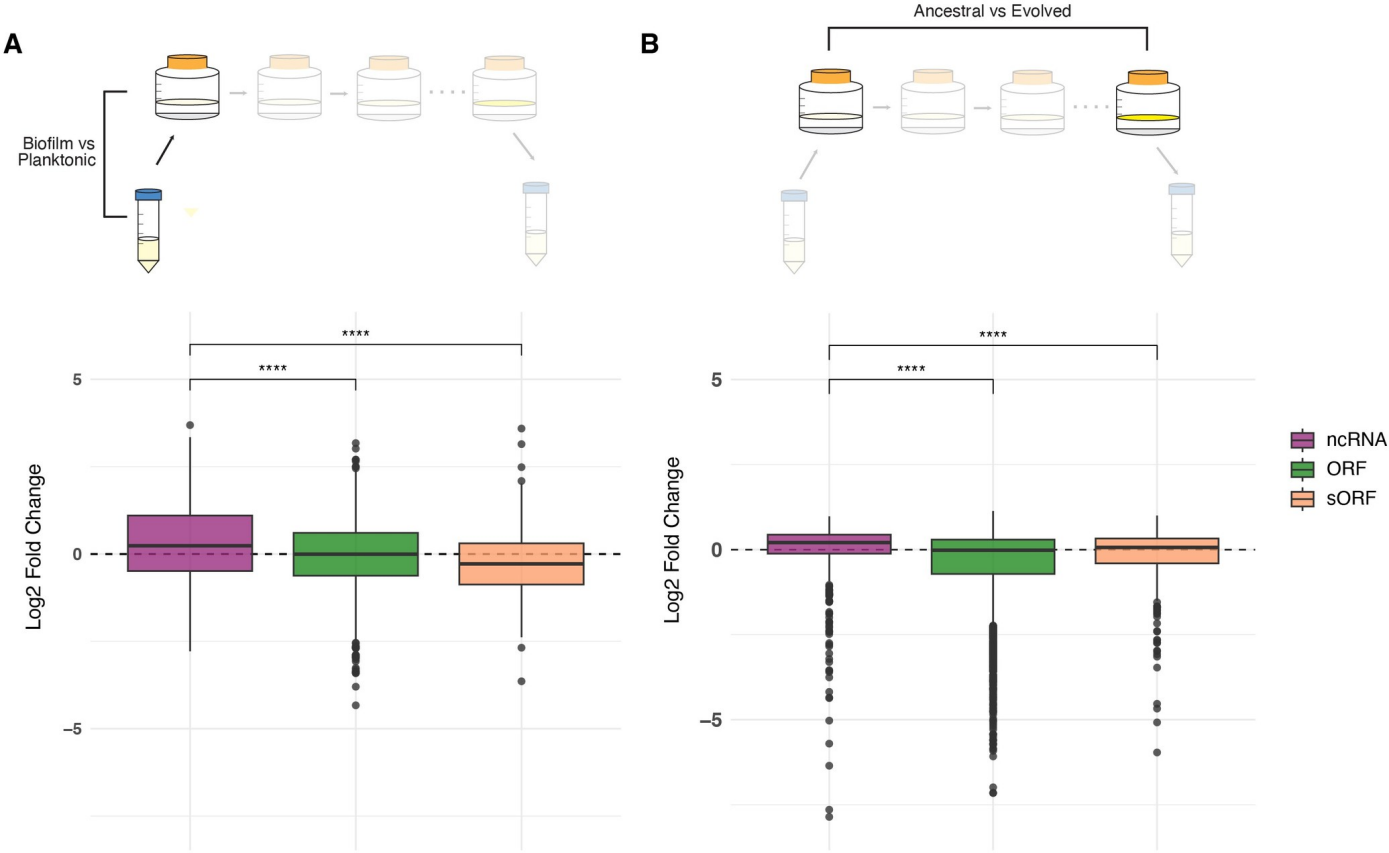
Upregulation of ncRNA is a common feature of *M*. *tb* biofilm growth. A) Log2 fold change (L2FC) values from ancestral populations by feature type. ncRNA have significantly higher L2FC values than ORFs and sORFs (Mann Whitney U Test with Benjamini-Hochberg correction, *p* < 0.0001) indicating they are more likely to be upregulated in biofilms. B) L2FC values between evolved and ancestral biofilms by feature type. ncRNA expression increased significantly more than either ORFs or sORFs after biofilm passage (Mann Whitney U Test with Benjamini-Hochberg correction, *p* < 0.0001).

One pattern that was particularly striking was the upregulation of ncRNAs in evolved biofilms: among the DEGs common to evolved populations (the evolved pellicle transcriptome), ncRNA represent 50% of all strongly upregulated features (L2FC > 2 in all populations) despite comprising only 12% of all features; they make up 4% of all strongly downregulated features (L2FC < -2 in all populations; S2 Data). Table 1 provides data for the most strongly differentially expressed ncRNAs in evolved pellicle populations (a cutoff of -5 was used for downregulated ncRNA to reduce the size of the table, all downregulated ncRNA can be found in S2 Data). Comparing the distribution of log2 fold change values of ncRNA with other feature types (ORFs and sORFs), we found that ncRNAs were significantly more likely to be upregulated in ancestral biofilms (Fig 8A), as well as being significantly more likely to be upregulated as a result of passaging (Fig 8B). Our results demonstrate that upregulation of ncRNA seems to be a common feature of the *M*. *tb* biofilm transcriptome, and suggests that non-canonical features such as small proteins and non-coding RNAs could play an important role in *M*. *tb* adaptation to new environments.

**Table 1:** Most significantly downregulated (< -5 L2FC) and upregulated (> 2 L2FC) ncRNAs in evolved populations grown as biofilms (compared to planktonic cultures). Features included in this list are significantly differentially expressed when all populations were analyzed together, as well as in at least five individual populations. Log2 fold change (L2FC) values given for analysis of all populations together.

Feature	L2FC	Upstream gene	Upstream gene L2FC	Downstream gene	Downstream gene L2FC
*ncRv10896*	3.15	*gltA2*	-3.31	*Rv0897c*	-0.72
*ncRv2240*	3.11	*Rv2240c*	1.77	*aceE*	-0.97
*ncRv3911Ac*	3.05	*sigM*	2.56	*ncRv3911Bc*	0.82
*ncRv1969c*	2.93	*mce3D*	1.96	*lprM*	1.59
*ncRv2379*	2.9	*mbtF*	1.24	*mbtE*	0.76
*ncRv0386c*	2.76	*Rv0386*	0.58	*purT*	0.86
*ncRv3480*	2.74	*Rv3480c*	-0.16	*Rv3481c*	1.67
*ncRv3507c*	2.73	*PE_PGRS53*	0.52	*PE_PGRS54*	-1.09
*ncRv3793c*	2.71	*embC*	0.88	*embA*	0.81
*ncRv2611*	2.69	*Rv2611c*	0.99	*pgsA1*	1.58
*ncRv1118*	2.54	*Rv1118c*	1.5	*Rv1119c*	0.36
*ncRv0664c*	2.48	*vapB8*	1.09	*vapC8*	0.94
*ncRv10186c*	2.32	*ncRv0186*	-0.63	*bglS*	-0.05
*ncRv1835A*	2.24	*Rv1835c*	1.08	*ncRv1835B*	0.1
*ncRv3917c*	-6.22	*parB*	-2.03	*Rv3916c*	-2.38
*ncRv12929*	-6.84	*Rv2929*	-0.73	*fadD26*	-3.93
*ncRv11221*	-7	*sigE*	-5.56	*rseA*	-2.55
*ncRv13460c*	-7.74	*rpsM*	-5.78	*rpmJ*	-5.74
*ssr*	-8.90	*Rv3099c*	-1.92	*Rv3098A*	-2.75
*ncRv1954*	-9.49	*Rv1954c*	0.57	*higB*	-3.67

## Discussion

Here we have characterized the diversity of transcriptomes among closely related clinical strains of *M*. *tb*. We observed genome-wide changes in gene expression in response to the application of a specific selective pressure, resulting in convergence on a biofilm transcriptome that was structured by lineage. Characterization of the pellicle transcriptome revealed a complex relationship between gene dosage, gene expression and the upregulation of non-coding RNA. Overall, this work highlights the complexity of the regulatory system in *M*. *tb* and provides a framework for considering the transcriptome as a target of selection. We have further identified novel candidate loci associated with *M*. *tb* pellicle formation.

### M. tb biofilm transcriptome: one size does not fit all

Little is known about the structure and function of *M*. *tb* biofilms [38], and even less about the transcriptional signatures that define this growth form. To our knowledge there is just one prior study of pellicle biofilm transcriptomics [16], which pinpointed a role for isonitrile lipopeptide (INLP) in biofilm growth. In their study of an attenuated strain of *M*. *tuberculosis* (mc^2^7000), Richards et al. (*16*) discovered that a 6-gene cluster termed the INLP synthase complex (*PPE1*, *Rv0097*, *fcoT*, *fadD10*, *Rv0100*, *nrp*) was highly upregulated during pellicle growth and in further experimental work they identified INLP in mycobacterial biofilms.

In the present study of closely related, fully virulent clinical strains of *M*. *tb*, we found that the pellicle biofilm transcriptome exhibited substantial variation among strains (Figs 2 and 3). This variation is evident in the number of genes differentially expressed under biofilm growth conditions (Fig 2), as well as the identity of genes subject to differential expression. As a result, there are few differentially expressed genes that are shared among strains (Fig 3C and 3D) and strain-to-strain variation dwarfs transcriptomic signals differentiating planktonic and biofilm growth (Fig 3B). Among this variation, we identified one strain that replicated the INLP finding of Richards et al (*16*). Four out of the six genes in the INLP synthase complex were upregulated and in the 98th percentile of differentially expressed genes for the strain MT55 (S1 Data). Genes in the complex were not upregulated to any significant degree in any of the other strains in our study. Integrating our results with those of Richards et al. (*16*), we conclude that natural populations of *M*. *tb* exhibit substantial variation in the regulatory mechanisms underlying biofilm growth, such that there appear to be multiple paths to this complex phenotype. In the case of INLP, careful experimental work in a lab-adapted, attenuated strain identified a molecular component of the pellicle biofilm that also appears to be important in a subset of clinical *M*. *tb* strains. Further research will be needed to determine if the diversity among *M*. *tb* biofilms is at the level of gene expression or gene products. For example, such work is needed to determine whether INLP is a universal component of the *M*. *tb* biofilm. Our results show that there is no singular *M*. *tb* pellicle transcriptome, even among closely related clinical strains: future studies should account for this natural diversity in identifying transcriptomic responses to biofilm and other growth conditions.

### Flattening and global repression in evolved populations

The application of a uniform selection pressure on the strains in our sample resulted in evolution of a more uniform transcriptome (S5B Fig), loss of strain-to-strain variation, and disambiguation of gene expression under conditions of biofilm growth (Fig 4). This shift suggests that the transcriptomic diversity found among *M*. *tb* strains in our study, prior to the imposition of selection in the laboratory, reflects their adaptation to distinct natural environments. The transcriptome that evolved under selection for pellicle growth is characterized by widespread repression of gene expression, a pattern that was evident in ancestral populations but markedly intensified after passaging (Figs 3C and 4C). Of the ~3600 genes differentially expressed across all pellicle passaged populations, there are similar numbers of down and upregulated genes (Fig 4C); however, the magnitude of the most downregulated genes exceeded 3x the magnitude of the most highly upregulated genes (S2 Data). Similar patterns of widespread repression have been identified previously, in studies of bacteria isolated from sputum [39,40] and in vitro models of dormancy [5]. The pellicle model differs from sputum and dormancy, however, in the global regulatory schemes that dictate the transcriptional program. The DosR regulon controls a group of genes that are activated under anaerobic conditions to trigger entry into a non-replicating dormant state [41]. Expression of the DosR regulon is a feature of the dormancy transcriptome [5] and has also been shown to characterize the sputum transcriptome [39,42,43]. Data from Richards et al. show that the DosR regulon was largely downregulated in their pellicle model, relative to exponential growth [16]. Here, we found that except for one strain (ancestral MT72), all ancestral and evolved biofilms showed significant downregulation of DosR regulon genes (S1 and S2 Data). Thus, it appears that while gene expression during pellicle growth shares some similarities to sputum and dormancy, the mechanisms of adaptation to pellicle growth appear to occur most commonly by a distinct, DosR-independent mechanism. These results also reinforce the importance of strain diversity in shaping transcriptomic responses to distinct environments as we again find that individual strains in our sample (in this case, ancestral MT72) employ distinct transcriptional strategies for biofilm growth. Recent research has shown that laboratory-induced dormancy phenotypes vary among strains of *M*. *tb* [44]. Together, these data show that transcriptional and phenotypic responses to environmental cues such as hypoxia vary within natural populations of *M*. *tb*.

The environmental cues used to induce pellicle formation include minimal media (Sauton’s), hypoxia and re-aeration, and prolonged culture. Here, as in Richards et al (*16*), we compared bacteria grown under pellicle conditions with those under standard planktonic conditions, which involves distinct media and duration of culture. Thus, some of the observed transcriptomic differences between pellicle and planktonic conditions may reflect bacterial responses to media and growth phase in addition to growth as a biofilm. In order to investigate potential overlaps in transcriptomic responses to culture media, growth phase and pellicle growth, we directly compared transcriptomic data generated under different conditions in H37Rv. Summary transcriptomics data from these experiments show that pellicle growth is readily distinguishable from culture in stationary phase and in minimal media (S8A Fig). Interestingly, bacterial responses to minimal media were characterized by even greater repression of gene expression than growth in stationary phase or a biofilm (S8B Fig). At the level of individual genes, we identified a group of upregulated elements, including *whiB7* and two non-coding RNAs, which were common to both stationary phase and pellicle growth in H37Rv (S2 Data). These elements were among the “evolved pellicle transcriptome”, i.e. among the DEGs commonly observed to differ in comparisons of evolved bacterial populations grown as pellicles and planktonically (exponential phase, 7H9 media, with detergent and shaking). This suggests that a subset of *M*. *tb*’s regulatory architecture can be recruited across stimuli and strains to respond to conditions found during prolonged culture. Given the transcriptional diversity of *M*. *tb* populations, it is possible that the extent of overlap between transcriptional responses to different stimuli varies from strain to strain. Future work investigating strain to strain diversity in *M*. *tb* transcriptional responses may illuminate more and less stable segments of *M*. *tb*’s regulatory architecture.

### Signatures of pellicle adaptation in the non-coding transcriptome

Growth as a biofilm was evident in the non-coding transcriptome, in addition to the coding transcriptome. Expression of non-coding RNAs (ncRNAs) was increased relative to that of genes during biofilm growth of ancestral populations (Fig 8A). Evolution under selective pressure for biofilm growth reinforced this pattern with ncRNA being significantly more upregulated than other loci after passaging (Fig 8B). Considering both ncRNAs and small open reading frames (sORFs), adaptation to pellicle growth resulted in emergence of a uniform biofilm transcriptome that was clearly distinguishable from planktonic conditions (Fig 7). We identified 14 ncRNAs that were significantly upregulated (L2FC > 2) and six that were significantly downregulated (L2FC < -5) in our pellicle passaged populations (Table 1). These are candidate loci mediating shifts in gene expression that enable *M*. *tb* growth as a biofilm: elucidating their specific functions will require further experimental work.

It has been shown that upregulation of even a single ncRNA can induce widespread gene repression in *M*. *tb* [26,29]. There are many targets of ncRNA regulation including genes involved in lipid metabolism [27], response to iron-limitation [29] and two-component regulatory systems (2CRS) [31]. Based on these studies, it has been hypothesized that in *M*. *tb*, ncRNA mediate a rapid response to new environmental stimuli, whereby changes in expression of a small number of ncRNA can have large impacts by regulation of many other genes. A similar phenomenon was recently uncovered in *C*. *difficile* where ncRNAs were shown to control initiation of sporulation [45]. We show here that ncRNAs are upregulated in our pellicle biofilm model and identify specific ncRNAs that are associated with biofilm adaptation. We hypothesize that modulation of ncRNAs contributes to the widespread changes in gene expression we see in pellicle passaged populations, a phenomenon we have termed Non-Coding Regulatory Reprogramming (NCRR). Further experimental work, likely integrated with computational modeling, will be needed to investigate the impacts of multiple simultaneous perturbations to *M*. *tb*’s complex regulatory network as we observed here. The ncRNAs and genes (Table 1 and S2 Data) identified as candidate biofilm loci can serve as a guide for research in this area. Experiments aimed at capturing very small RNAs (smaller than the ~200-300nt minimum included in this study) may further illuminate regulatory mechanisms of *M*. *tb* biofilm growth.

### How do transcriptomes evolve?

Experimental evolution studies have shown that many adaptive mutations in bacteria are predicted to have a regulatory effect. Studies of *Escherichia coli* [46–48] and *Pseudomonas aeruginosa* [49] indicate that rapid adaptation to new environments can be accomplished with a few well-placed mutations in regulatory genes, a pattern we observed in our experiment evolving *M*. *tb* [17]. What is less well-known however, is the impact of these mutations on the transcriptional landscape. We sought to characterize the effects of these adaptive mutations on the transcriptome. We found that selection for pellicle growth resulted in stronger downregulation of genes (Figs 3C and 4C), reduction of sample-to-sample diversity (Figs 3B and 4B) and further upregulation of ncRNA (Fig 8B), relative to ancestral populations. Of particular interest is the reduction in diversity between biofilm transcriptomes after passaging, an indication that these populations converged onto a shared transcriptome under the same selective pressure. These findings extend observations from previous studies illustrating parallel transcriptome adaptation after imposition of a uniform selection pressure. Under laboratory conditions, convergence towards a shared genic transcriptome has been observed several times in bacteria [49–52] and even in fungi [53]. One study of *P*. *aeruginosa* transcriptomes from an explanted lung showed that genetically distinct strains exhibited minimal variability in their transcriptomes [54], indicating that this phenomenon likely holds true for bacterial adaptation during infection. Here, we have shown that the phenomenon applies to *M*. *tb*, and that it also extends to the non-coding transcriptome.

We identified a striking phenomenon beyond this convergence on a shared transcriptome. Our findings suggest that the adaptive trajectories of both the coding and non-coding transcriptomes are shaped by the genetic background of the bacterial population (Figs 5A and 7B). Populations with differentiated transcriptomes at baseline evolved shared, lineage-specific transcriptomes under selection for biofilm growth. This pattern was specific to biofilm growth, as neither convergence nor an effect of genetic background were evident during planktonic growth of evolved populations (Figs 5B and S7). In our prior study describing genomic adaptations during this experiment, we found evidence that strain genetic background affects the types of mutations observed during selection for pellicle growth [17]. Convergence on a strain-specific transcriptome could thus reflect the mutational trajectory associated with each genetic background, since the majority of observed mutations appeared to act by a regulatory mechanism [17]. We discuss this possibility in more detail below.

### Transcriptomic consequences of mutations

One of the mutations that exhibited an association with lineage is a 175 kb duplication we termed the MMMC duplication (Roman numeral 3100, an umbrella term for duplications starting at *Rv3100)*. The MMMC duplication has been observed previously, and structural variants appear to be common in *M*. *tb* populations, likely facilitated by recombination among IS elements [22]. We previously found the MMMC duplication to be specifically associated with pellicle adaptation and the L4.9 background. Here, we found that *M*. *tb* populations from the L4.9 background, known to have developed the MMMC duplication, converged on a similar transcriptome (Fig 5A).

The evolutionary history of *M*. *tb* is littered with evidence of structural variants, particularly duplications, which have formed some of the most important gene families such as the Type VII secretion systems [55] and PE/PPE genes [56]. In addition to the creation of new gene functions, duplications are also known to increase fitness through gene dosage effects. For example, an attenuated strain of *M*. *tb* missing ESX-3 effectors *esxG* and *esxH* developed duplications of a set of paralogous effectors which, when overexpressed as a result of duplication, compensate for the loss of *esxG* and *esxH* [57]. Despite their prevalence in *M*. *tb*, transcriptional impacts of large structural variants are not well understood. A prior study of a genomic deletion found that it was associated with loss of expression of genes within the deletion [58], but to our knowledge there are no prior studies investigating transcriptional impacts of large duplications. Here we sought to identify the transcriptional impacts of the MMMC duplication. We identified a two-fold increase in gene dosage from the MMMC [17], thus we might expect the duplication to result in a two-fold increase in expression of genes within the duplication. However, this was not the case. Effects of the duplication on gene expression appeared to be highly variable, with some genes exhibiting expected two-fold increases (log2 fold change of 1) and others with as much as 9-fold increases (log2 fold change of ~3) and 50-fold decreases (log2 fold change of ~ -5) (Fig 6C and S4 Data). These findings illuminate a complex relationship between mutation, gene expression, and phenotype that belies a simple one to one map between gene dose and gene expression.

Another potential mechanism underlying this complex pattern is that the duplication of such a large region in the genome interrupts the function of regulatory factors that are either encoded within, or act on loci within the duplication. Duplication of binding sites for a regulatory factor outside the duplicated region could ‘dilute’ the effect of that regulator. Conversely, increased gene dosage of regulators within the duplication could affect expression of genes within and outside the duplication. As an initial step toward investigating this hypothesis, we examined the data for impacts of increases (encoded within the duplication) and decreases (binding within the duplication) in transcription factor dosage, based on published transcription factor overexpression studies [25]. Our data did not conform with predicted impacts of changes in transcription factor dosage. We hypothesize that the compounded effects of increased dosage of multiple factors within a complex regulatory architecture may have far-reaching, and non-linear impacts on gene expression following large-scale duplication events. Further analysis of the specific regulators, particularly non-coding RNAs, and regulatory binding sites within the MMMC duplication may help to explain observed transcriptomic impacts of the duplication.

Whatever the mechanism of MMMC’s impacts on gene expression, emergence of the duplication was associated with convergence on a lineage-specific transcriptome (Fig 5A). This could suggest that similarities in emergent transcriptomes arise from the mutation itself and the lineage-specificity of the evolved transcriptome simply reflects a mutational bias for these strains. However, strains from lineage 4.4 also converged on a similar transcriptome (Figs 5A and 7B) without evolving a mutation in common [17]. We also observed the MMMC outside of lineage 4.9, as an apparently transient mutation in MT49 (S1 Text), showing that the mutation is not specific to the L4.9 strains in our sample. While the effects of the duplication on MT49 were evident in expression of genes within its borders, overall adaptation of the transcriptome followed a similar trajectory to other strains from L4.4, which lacked the duplication. As noted above, our ancestral populations demonstrated strain-to-strain variability in transcriptomic responses to biofilm growth. Prior research has also described transcriptomic variation among *M*. *tb* isolates [59–61]. Based on our observations here and prior research, we hypothesize that *M*. *tb* populations are capable of rapidly adapting their transcriptomes to new environments, likely enabled by flexible deployment of ncRNAs, but that this adaptation is constrained by genetic background. Thus, we posit that *M*. *tb* transcriptome diversity reflects both canalization (the tendency of a population to produce the same phenotype regardless of genotype) arising from impacts of lineage structure on regulatory networks, as well as variation in selection pressures encountered during natural infection.

### Implications & future directions

We know that biofilms represent heterogeneous populations, with sub-populations that differ in genotype and functional specialization [62]. Recent work using spatial transcriptomics has also begun to reveal the ways in which bacterial communities coordinate gene expression to form biofilms [63,64]. Our analyses in this work have compressed the diversity of the *M*. *tb* biofilm transcriptome within this population to a single dimension. Future work studying the diversity of gene expression within biofilm sub-populations will give us more insight into the form and function of *M*. *tb* biofilms.

In this study we have characterized both the patterns of gene expression within *M*. *tb* biofilms, as well as how those patterns change upon the application of a complex selective pressure. We discovered a cadre of upregulated ncRNA that we hypothesize to be involved in genome-wide gene expression modulation. Further experimental work will be needed to define the specific roles of candidate biofilm-associated loci from this study, including genes, regulators and ncRNAs, as well as their coordinated impacts on overall gene expression. We have also illustrated the complex effects on expression of large structural variants. These results give us valuable insight into how *M*. *tb* adapts to new environments. The complexity of the selection pressure applied in this experiment, as well as our focus on characterizing different strains of *M*. *tb* make these results particularly relevant to the study of natural infections and adaptation of *M*. *tb* under within-host selection pressures.

## Materials and methods

### Bacterial strains and growth conditions

Six clinical populations (MT31, MT49, MT55, MT72, MT345, and MT540) were initially isolated from sputum samples and passaged repeatedly as pellicle biofilms as previously described [17]. Ancestral (prior to biofilm passaging, from frozen stocks of low-passage clinical isolates) and evolved (after biofilm passaging, from frozen stocks made during the initial experiment) populations were grown both as planktonic cultures and biofilms for RNA sequencing. Planktonic cultures of *M*. *tuberculosis* were inoculated from freezer stocks into 7H9OTG (Middlebrook 7H9 broth [HiMedia, #M198] containing 0.2% w/v glycerol, 10% v/v OADC supplement [oleic acid, albumin, D-glucose, catalase; Becton Dickinson, # B12351] and 0.05% w/v Tween-80) and incubated at 37°C with 5% CO_2_ to an OD600 ~1 before RNA extraction. For biofilm growth, 250 μL of planktonic culture at an OD600 of 1 was inoculated into 25mL Sauton’s medium (for 1L: 0.5g KH_2_PO_4_, 0.5g MgSO_4_, 4g L-Asparagine, 2g Citric acid, 0.05g Ammonium Iron (III) citrate, 60mL glycerol, adjust pH to 7.0 with NaOH) containing 0.1% w/v ZnSO_4_, in a 250 mL flask (Corning, #430281) and incubated at 37°C, with 5% CO_2_, without shaking, with a tight-fitting cap. After 3 weeks the lid was loosened to allow gas exchange and the cultures grown for an additional 2 weeks (for a total of 5 weeks of growth) before RNA extraction.

For the comparison of growth phase, evolved MT31 and H37Rv were seeded into 7H9OTG and grown in planktonic culture as described above to exponential (OD600 ~1, ~5 days) and stationary (9 days) phases before harvest. To compare the effect of media on the transcriptome H37Rv was grown under planktonic conditions in Sauton’s with 0.05% w/v Tween-80 for 9 days. H37Rv biofilm cultures in Sauton’s were performed as described above.

### RNA extraction

Biofilm samples were harvested by first removing and discarding the media below the biofilm. The biofilm was resuspended in fresh Sauton’s medium, pelleted at 5,000 xg for 10 minutes, then resuspended in 3 mL of RNAprotect for bacterial cells (Qiagen, #76506). Planktonic cultures were similarly pelleted and resuspended in RNAprotect. Aliquots (biological replicates) of the RNAprotect suspensions were frozen at -80°C before extraction. Before RNA extraction, RNAprotect suspensions were thawed and pelleted at 5,000 xg for 10 minutes before discarding the supernatant. The pellet was then resuspended in 100 μL of 100 mg/mL lysozyme (Sigma, #L7651) and 150 μL of Proteinase K (Qiagen, #19131), vortexed, and incubated at 37°C for 30 minutes (vortexed every 10 minutes). The remainder of the extraction was performed with a modified version of the Illustra RNAspin mini RNA isolation kit (GE Healthcare, #25-0500-71) protocol: 350 μL of buffer RA1, 3.5 μL of *β*-mercaptoethanol (Sigma, #M6250) and 25–50 mg of acid-washed glass beads (Sigma, #G8772) were added to the cell suspension. Tubes were vortexed at top speed for a total of 3 minutes (6 x 30 sec vortexes with 1 minute ice rest between vortexes) and then centrifuged at 11,000 xg for 1 minute. The supernatant was removed before proceeding with step 3 of section 7.3 of the kit protocol. RNA quantification was performed by Qubit using the RNA HS Assay Kit (Invitrogen, #Q32852).

### Library preparation & sequencing

Total RNA submitted to the University of Wisconsin-Madison Biotechnology Center was assayed for purity and integrity via the NanoDrop One Spectrophotometer and Agilent 2100 Bioanalyzer, respectively. For library preparation, ribosomal RNA was removed from total RNA using the RiboMinus Eukaryote System v2 (ThermoFisher, #A15026) kit following the directions provided by the manufacturer. Using the TruSeq Stranded RNA kit (Illumina, Inc., Carlsbad, CA), the ribo-depleted RNA was fragmented using divalent cations under elevated temperature. Fragmented RNA was copied into first stranded cDNA using SuperScript II Reverse Transcriptase (Invitrogen, Carlsbad, California, USA) and random primers. Second strand cDNA was synthesized using a modified dNTP mix (dTTP replaced with dUTP), DNA Polymerase I, and RNase H. Double-stranded cDNA was cleaned up with AMPure XP Beads (1.8X) (Agencourt, Beckman Coulter). The cDNA products were incubated with Klenow DNA Polymerase to add a single ‘A’ nucleotide to the 3’ end of the blunt DNA fragments. Unique dual indexes (UDI) were ligated to the DNA fragments and cleaned up with two rounds of AMPure XP beads (0.8X). Adapter ligated DNA was amplified by PCR and cleaned up with AMPure XP beads (0.8X). Final libraries were assessed for size and quantity using an Agilent DNA1000 chip and Qubit dsDNA HS Assay Kit (Invitrogen, Carlsbad, California, USA), respectively. Libraries were standardized to 2nM. Paired-end 150bp sequencing was performed, using standard SBS chemistry on an Illumina NovaSeq6000 sequencer. Images were analyzed using the standard Illumina Pipeline, version 1.8.2.

For H37Rv and evolved MT31 grown for the analysis of media composition and growth phase on the transcriptome, RNA was submitted to SeqCoast for library preparation and sequencing. Briefly, samples were prepared for sequencing using an Illumina Stranded Total RNA Prep Ligation with Ribo-Zero Plus Microbiome and unique dual indexes. Sequencing was performed on the Illumina NextSeq2000 platform using a 300 cycle flow cell kit to produce 2x150bp paired reads.

### Custom annotation file

A custom annotation file (available at github.com/myoungblom/mtb_ExpEvo_RNA) was made using the standard annotations for reference strain H37Rv (NCBI accession GCA_000195955.2). To these annotations we added non-coding RNA (ncRNA) compiled from recent publications [26–32] as well as data provided by the Fortune lab [[29]; available under NCBI BioProject number PRJNA451488]. Finally we included a subset of the small open reading frames (sORFs) discovered by C. Smith et al., 2021: only sORFs identified in multiple technical replicates using Ribo-RET, that were antisense to or not overlapping any other genes were included [65]. The resulting annotation file contains a total of 579 ncRNA and 300 sORFs (S5 Data).

### RNA sequencing data analysis

Raw RNA-sequencing data was processed using an in-house pipeline (code available at github.com/myoungblom/RNAseq). Briefly, raw data was checked for quality with FastQC v0.11.8 (http://www.bioinformatics.babraham.ac.uk/projects/fastqc) and trimmed using Trimmomatic v0.39 [66]. Trimmed reads were mapped to the H37Rv reference genome (NCBI accession NC_000962.3) using BWA-MEM v0.7.17 [67], and Samtools v1.17 [68] view and sort were used to process SAM and BAM files. Assembly quality was determined using Qualimap v2.2.1 BamQC and RNAseq tools [69]. Finally, gene expression was counted using HTSeq counts v1.99.2 [18] using the custom annotation file described above with flags ‘—nonunique none’ and ‘-s reverse’ to exclude reads mapping to more than one feature and to indicate a reverse-stranded library prep, respectively.

### Differential expression analysis

Differential expression analysis was performed in R v4.2.2 [70] using DESeq2 v1.30.0 [19]. First, gene expression counts from HTSeq were loaded using the function ‘DESeqDataSetFromHTSeqCount’, and genes with ‘0’ counts in all samples were removed. A variance stabilizing transformation was applied to the raw expression counts using the function ‘vst’ which additionally normalizes counts with respect to library size–these counts were used for visualizations including PCA plots and heatmaps. Significantly differentially expressed genes (DEGs) were identified for each comparison using the function ‘DESeq’, filtering results by *p*-value (with Benjamini-Hochberg correction) of < 0.05. For analyses of all populations, all samples were analyzed with DESeq together using either the growth condition (biofilm or planktonic) or the genotype (evolved or ancestral) as the design factor. For analyses of individual populations, only samples from a given population were included in the DESeq analysis, using the same design criteria as listed above. Scripts for differential expression analyses and figures are available here: github.com/myoungblom/mtb_ExpEvo_RNA.

### Phylogenetic tree

Phylogenetic tree of ancestral populations was inferred using RAxML v8.2.3 as described previously [17] and plotted using ggtree [71].

### Sliding coverage plots

Sliding window DNA and RNA sequencing coverage was calculated from sorted BAM files created from the mapping pipeline described above, using Samtools bedcov [68] with a window size of 20,000 bp and a step size of 5,000 bp (code available at github.com/myoungblom/mtb_ExpEvo). Relative coverage was calculated by dividing each window coverage by the average coverage across the assembly–as calculated by Qualimap BamQC [69].

## Supporting information

S1 Text Transcriptomic impacts of biofilm-associated mutations. (DOCX)

S1 Table RNA sequencing sample information. Passage number 1 refers to the ancestral population and evolved populations were sequenced after either 8 or 12 passages. Number of mapped reads and percentage of reads assigned to a feature were calculated using Qualimap v2.2.1 [69].
(DOCX)

S1 Fig No batch effects observed for library preparation or sequencing batch. Principal component analysis (PCA) of variance stabilizing transformed gene expression counts for all samples, colored by library preparation batch (A) or sequencing batch (B). Resequencing refers to additional sequencing of some samples to achieve target sequencing depth.
(PNG)

S2 Fig High concordance of biological replicates. Pairwise distance values calculated from variance stabilizing transformed expression counts between samples that are not biological replicates (NO) and samples that are biological replicates (YES). Biological replicates have significantly shorter distances (Mann Whitney U Test, *p* = 3e-5) than non biological replicates with two outliers: MT31 evolved planktonic (*) and MT72 ancestral biofilm (**) samples show discordance between their biological replicates which we took into consideration in the analysis of our differential expression results.
(PNG)

S3 Fig A) Principal component analysis (PCA) of variance stabilizing transformed gene expression counts from ancestral populations. Each point represents the total gene expression of a single sample, where the shape indicates the growth condition of the sample, and the color indicates the population. B) PCA (same as in A) colored this time by biofilm wet weight. There is no clear correlation–either among biofilm samples or planktonic samples–between gene expression patterns and ancestral biofilm phenotype (as measured by wet weight).
(PNG)

S4 Fig Moderate differential expression under biofilm conditions for ancestral populations. A zoomed in version of Fig 3C.
(PNG)

S5 Fig Evolved biofilm populations have more similar transcriptomes. A) Principal component analysis (PCA) of variance stabilizing transformed gene expression counts from evolved populations. Each point represents the total gene expression of a single sample, where the shape indicates the growth condition of the sample, and the color indicates the population. B) Pairwise distance values calculated from variance stabilizing transformed expression counts between samples of the same genotype (ancestral or evolved) and the same growth condition (biofilm or planktonic). Evolved biofilm populations have significantly shorter distances between samples (Mann Whitney U Test with Benjamini-Hochberg correction, *p* = 1.5e-15) than their ancestral counterparts, indicating more similar gene expression profiles after passaging. Conversely, populations grown in planktonic culture have significantly higher inter-sample distance after passaging (Mann Whitney U Test with Benjamini-Hochberg correction, *p* = 7.4e-3) indicating that diversity of gene expression under planktonic conditions is unaffected by biofilm passaging. Distances between biological replicates have been excluded.
(PNG)

S6 Fig Heatmaps of variance stabilizing transformed expression counts all genes in the genome. Each column is a single sample from an ancestral (A) or evolved (B) population, grown either as a biofilm or in a planktonic culture. Each row is a gene. Heatmap colored by expression values for each gene which are normalized to the mean across samples. Samples are clustered by Euclidean distance and plotted as a tree at the top of the heatmap. Evolved populations have much more uniform biofilm transcriptomes.
(PNG)

S7 Fig Treating evolved MT31 outlier as a biofilm. A) Top: Experimental diagram highlighting comparator populations: evolved populations grown as pellicle biofilms are compared to the same populations grown in planktonic cultures. Bottom: Principal component analysis (PCA) of variance stabilizing transformed gene expression for evolved populations grown as biofilms and planktonic cultures. B) Differential expression of DEGs between evolved populations grown as biofilms and as planktonic cultures. Log transformed adjusted p-values plotted against the log2 fold change for each gene. Genes that did not have significant differential expression are shown in grey. C) Heatmap of normalized, variance stabilizing transformed expression counts for DEGs shown in panel B. Each column is a single sample from an evolved population, grown either as a biofilm or in a planktonic culture–the sample identified as an outlier in Fig 4 is highlighted by an arrow. Each row is a DEG. Expression values for each gene are normalized to the mean across samples. Samples are clustered by Euclidean distance and plotted as a tree at the top of the heatmap. D) Matrix of individual DEGs shared across evolved populations. A total of 4373 DEGs are plotted according to if that gene is upregulated (red), downregulated (blue) or not significantly differentially expressed (white) in each population. 24% of downregulated genes are shared by at least 5 populations (*), while 16% of upregulated genes are shared by at least 5 populations (**).
(PNG)

S8 Fig Transcriptomes of pellicles versus stationary phase and growth in minimal media. A) Principal component analysis (PCA) of variance stabilizing transformed gene expression for H37Rv grown in different media, and under different growth conditions. Patterns of gene expression associated with four conditions (minimal media, stationary phase, exponential phase, pellicle biofilm) were distinct. B) Counts of highly differentially expressed genes (DEGs, Log2 fold-change >2/<-2) for H37Rv in pellicle biofilms, Sauton’s minimal media, and stationary phase, relative to exponential phase planktonic culture. Counts of DEGs shared among conditions are also shown. C) Highly differentially expressed genes (DEGs, Log2 fold-change >2/<-2) for evolved MT31 in stationary phase vs planktonic, and pellicle biofilms vs planktonic culture. No DEGs were common to both conditions.
(PNG)

S9 Fig A) Log-2 fold changes (L2FC) for all genes in each biofilm population, separated by their presence in one or both duplications acquired by MT31 and MT55. Mann Whitney U test with Benjamini-Hochberg correction shows significantly higher L2FC values for duplicated genes in MT31 and MT55 (same data shown in Fig 6C), and significantly lower L2FC values for duplicated genes in MT49 and MT72. Comparison is between evolved populations grown as a biofilm and ancestral populations grown as biofilms. B) Same as A, but comparing pellicle evolved populations grown as planktonic cultures, to ancestral populations grown as planktonic cultures. Mann Whitney U test with Benjamini-Hochberg correction shows significantly higher L2FC values for duplicated genes in MT31 and MT55 and significantly lower L2FC values for duplicated genes in MT49.
(PNG)

S10 Fig A) log2 fold change (L2FC) values between evolved and ancestral biofilm populations of MT49. Shown is each gene within the region surrounding the duplication which arose in MT31 and MT55. Points are colored according to whether they lie inside of the duplicated region. B) Top: Relative coverage of DNA sequencing for the ancestral (passage 0) and evolved (passage 12) populations of MT49. Bottom: Relative coverage of RNA sequencing for ancestral and evolved populations of MT49. Two lines per genotype indicate two biological replicates.
(PNG)

S11 Fig A) Schematic of *lpdA* operon relative to the two intergenic SNPs acquired by MT49 and MT540 over the course of passaging. Genes are colored by functional annotation from Mycobrowser: orange–Intermediary metabolism and respiration, purple–regulatory proteins, pink–conserved hypotheticals, green—cell wall and cell wall processes. B) Log-2 fold change (L2FC) in expression comparing evolved to ancestral populations, for genes downstream of a convergent intergenic mutation in two of our populations (MT49 and MT540). Points circled in black indicate significant L2FCs in expression between evolved and ancestral populations. ‘Biofilm’ refers to evolved populations grown as a biofilm, compared to ancestral populations grown as a biofilm, and the same is true for ‘planktonic’ as shown in diagrams at the top of the panel.
(PNG)

S12 Fig Principal component analysis (PCA) of variance stabilizing transformed expression counts of ncRNAs and sORFs from ancestral and evolved populations grown in planktonic culture. Arrows are drawn between corresponding ancestral and evolved populations, indicating the trajectory of evolution across passaging. Points are colored by sub-lineage of the ancestral population. Note that we do not know the exact evolutionary trajectory of these populations, these lines are merely a graphical representation of that trajectory.
(PNG)

S1 Data DEGs comparing ancestral populations grown as biofilms, to the same populations grown under planktonic conditions. DEGs provided for multiple analyses: all populations and each population individually, except for MT49 which has been removed because only one biological replicate was obtained under planktonic conditions. Note that low concordance between biological replicates of ancestral MT72 grown under biofilm conditions may impact the results of these analyses. “SharedDEGs” sheet refers to features which were significantly differentially expressed when all populations were analyzed together, as well as in at least four (MT49 was excluded) individual populations and have been annotated with their functional category.
(XLSX)

S2 Data DEGs comparing pellicle passaged (evolved) populations grown as biofilms, to the same populations grown under planktonic conditions. DEGs provided for multiple analyses: all populations and each population individually. “SharedDEGs” sheet refers to features which were significantly differentially expressed when all populations were analyzed together, as well as in at least five individual populations and have been annotated with their functional category.
(XLSX)

S3 Data DEGs of H37Rv grown planktonically, as a pellicle biofilm, and in Sauton’s (minimal) media. (XLSX)

S4 Data DEGs comparing pellicle passaged (evolved) populations grown as biofilms, to ancestral populations grown as biofilms. DEGs provided for multiple analyses: all populations, each population individually and for each of the two sub-lineages in our sample (L4.4 and L4.9). Note that low concordance between biological replicates of evolved MT31 grown under planktonic conditions may impact the results of these analyses.
(XLSX)

S5 Data Non-coding RNAs (ncRNAs) and small open reading frames (sORFs) included to make the custom annotation file. (XLSX)
