# Supplementary material for: Adaptation of the Mycobacterium tuberculosis transcriptome to biofilm growth
Source: PLoS Pathog. 2024 Apr 18;20(4):e1012124. doi: 10.1371/journal.ppat.1012124 (PMC11060545; doi:10.1371/journal.ppat.1012124)
Supplement: S1 Text — (DOCX) [file ppat.1012124.s001.docx]

**Supplementary Text**

Transcriptomic impacts of biofilm-associated mutations

We expected expression changes of genes within the MMMC duplication to be specific to evolved populations of MT31 and MT55. However, we found two other populations with expression changes within this region: expression was overall downregulated in evolved populations of MT49 and MT72 under biofilm conditions (Figure S9A) and in MT49 under planktonic conditions (Figure S9B). The difference was relatively subtle for MT72 but striking for MT49 (Figure S10A). Coverage plots from DNA sequencing reads did not reveal a genomic deletion in this region for MT49 (Figure S10B). Using coverage of RNA sequencing in this region we observed that the ancestral population had relatively high expression of genes in this region compared to the surrounding area (Figure S10B). Thus, it appears that the difference in gene expression at this locus arises from increased expression in the ancestral population, rather than decreased expression in the evolved population. Our hypothesis is that a transient MMMC duplication arose in the ancestral population during the process of growing the population as a biofilm for RNA extraction and sequencing. This is further supported by RNA coverage of ~1.25x within this region indicating that the duplication was present at intermediate frequency when the sample was sequenced (Figure S10B). These data suggest that tandem duplications occur commonly, but that they are positively selected under specific conditions.

The MMMC mutation appeared specifically in association with biofilm selection on the MT31 and MT55 backgrounds. In another instance of convergent adaptation, L4.4.1.1 strains MT49 and MT540 both acquired intergenic SNPs upstream of *lpdA* (Figure S11A). We previously showed using quantitative PCR (qPCR) that these intergenic SNPs led to increased expression of the downstream gene (*lpdA*), likely by interfering with a known transcription factor binding site in this intergenic region [1]. Here we used the transcriptomics data to examine the rest of the genes in the operon downstream of *lpdA*, a total of six genes. We found that that the intergenic SNPs had variable effects on expression of downstream genes under biofilm conditions with some genes upregulated (*lpdA, glpD2, Rv3300c* and *lpqC*) while others were downregulated (*phoY1* and *atsB*), indicating that these genes are not all co-transcribed (Figure S11). Because this operon lies within the bounds of the MMMC duplication the analysis of MT49 under biofilm conditions was also confounded by the transient duplication as we described above for the MMMC duplication (Figure S11). Like the duplication these SNPs appear in association with biofilm growth and appear to have complex effects on gene expression.

1. Smith TM, Youngblom MA, Kernien JF, Mohamed MA, Fry SS, Bohr LL, et al. Rapid adaptation of a complex trait during experimental evolution of Mycobacterium tuberculosis. Soldati-Favre D, Ojha A, editors. eLife. 2022;11: e78454. doi:10.7554/eLife.78454
