# Supplementary material for: Adaptation of the Mycobacterium tuberculosis transcriptome to biofilm growth
Source: PLoS Pathog. 2024 Apr 18;20(4):e1012124. doi: 10.1371/journal.ppat.1012124 (PMC11060545; doi:10.1371/journal.ppat.1012124)
Supplement: S1 Table — Passage number 1 refers to the ancestral population and evolved populations were sequenced after either 8 or 12 passages. Number of mapped reads and percentage of reads assigned to a feature were calculated using Qualimap v2.2.1 [69]. (DOCX) [file ppat.1012124.s002.docx]

**S1 Table:** RNA sequencing sample information. Number of mapped reads and percentage of reads assigned to a feature were calculated using Qualimap v2.2.1(Okonechnikov et al., 2016) [70].

| **Sample Number** | **Population** | **Passage Number** | **Condition** | **RNA sequenced (ug)** | **Mapped reads (millions)** | **Reads assigned to feature (millions)** |
| --- | --- | --- | --- | --- | --- | --- |
| 1 | MT31 | 0 | Planktonic | 1.29 | 73 | 66 |
| 2 | MT31 | 0 | Planktonic | 1.62 | 55 | 50 |
| 3 | MT31 | 12 | Planktonic | 7.04 | 62 | 36 |
| 4 | MT31 | 12 | Planktonic | 1.58 | 53 | 50 |
| 5 | MT31 | 0 | Biofilm | 0.95 | 51 | 32 |
| 6 | MT31 | 0 | Biofilm | 0.98 | 63 | 55 |
| 7 | MT31 | 12 | Biofilm | 2.64 | 79 | 54 |
| 8 | MT31 | 12 | Biofilm | 3.18 | 99 | 39 |
| 10 | MT49 | 0 | Planktonic | 1.52 | 69 | 64 |
| 11 | MT49 | 12 | Planktonic | 3.42 | 76 | 73 |
| 12 | MT49 | 12 | Planktonic | 2.49 | 69 | 56 |
| 13 | MT49 | 0 | Biofilm | 1.54 | 52 | 30 |
| 14 | MT49 | 0 | Biofilm | 3.21 | 117 | 59 |
| 15 | MT49 | 12 | Biofilm | 2.19 | 70 | 34 |
| 16 | MT49 | 12 | Biofilm | 0.95 | 83 | 29 |
| 17 | MT55 | 0 | Planktonic | 1.5 | 69 | 64 |
| 18 | MT55 | 0 | Planktonic | 0.82 | 54 | 50 |
| 19 | MT55 | 8 | Planktonic | 0.44 | 64 | 57 |
| 20 | MT55 | 8 | Planktonic | 3.4 | 75 | 72 |
| 21 | MT55 | 0 | Biofilm | 0.39 | 73 | 70 |
| 22 | MT55 | 0 | Biofilm | 7.33 | 82 | 81 |
| 23 | MT55 | 8 | Biofilm | 1.15 | 75 | 31 |
| 24 | MT55 | 8 | Biofilm | 1.40 | 77 | 32 |
| 25 | MT72 | 0 | Planktonic | 3.15 | 43 | 38 |
| 26 | MT72 | 0 | Planktonic | 1.89 | 62 | 54 |
| 27 | MT72 | 8 | Planktonic | 1.07 | 70 | 58 |
| 28 | MT72 | 8 | Planktonic | 0.72 | 55 | 52 |
| 29 | MT72 | 0 | Biofilm | 1.73 | 52 | 49 |
| 30 | MT72 | 0 | Biofilm | 4.22 | 63 | 54 |
| 31 | MT72 | 8 | Biofilm | 2.24 | 64 | 39 |
| 32 | MT72 | 8 | Biofilm | 1.16 | 54 | 33 |
| 33 | MT345 | 0 | Planktonic | 0.60 | 71 | 58 |
| 34 | MT345 | 0 | Planktonic | 1.14 | 72 | 69 |
| 35 | MT345 | 8 | Planktonic | 1.8 | 73 | 71 |
| 36 | MT345 | 8 | Planktonic | 0.87 | 51 | 49 |
| 37 | MT345 | 0 | Biofilm | 1.05 | 87 | 35 |
| 38 | MT345 | 0 | Biofilm | 1.32 | 87 | 28 |
| 39 | MT345 | 8 | Biofilm | 0.85 | 68 | 28 |
| 40 | MT345 | 8 | Biofilm | 1.2 | 90 | 34 |
| 41 | MT540 | 0 | Planktonic | 0.54 | 69 | 58 |
| 42 | MT540 | 0 | Planktonic | 1.56 | 71 | 68 |
| 43 | MT540 | 8 | Planktonic | 2.73 | 51 | 48 |
| 44 | MT540 | 8 | Planktonic | 2.88 | 72 | 65 |
| 45 | MT540 | 0 | Biofilm | 2.67 | 76 | 74 |
| 46 | MT540 | 0 | Biofilm | 1.91 | 61 | 59 |
| 47 | MT540 | 8 | Biofilm | 1.07 | 63 | 20 |
| 48 | MT540 | 8 | Biofilm | 1.46 | 80 | 33 |

References

70. K. Okonechnikov, A. Conesa, F. García-Alcalde, Qualimap 2: advanced multi-sample quality control for high-throughput sequencing data. Bioinformatics 32, 292–294 (2016).
