# Supplementary figures and images for: Adaptation of the Mycobacterium tuberculosis transcriptome to biofilm growth
Source: PLoS Pathog. 2024 Apr 18;20(4):e1012124. doi: 10.1371/journal.ppat.1012124 (PMC11060545; doi:10.1371/journal.ppat.1012124)

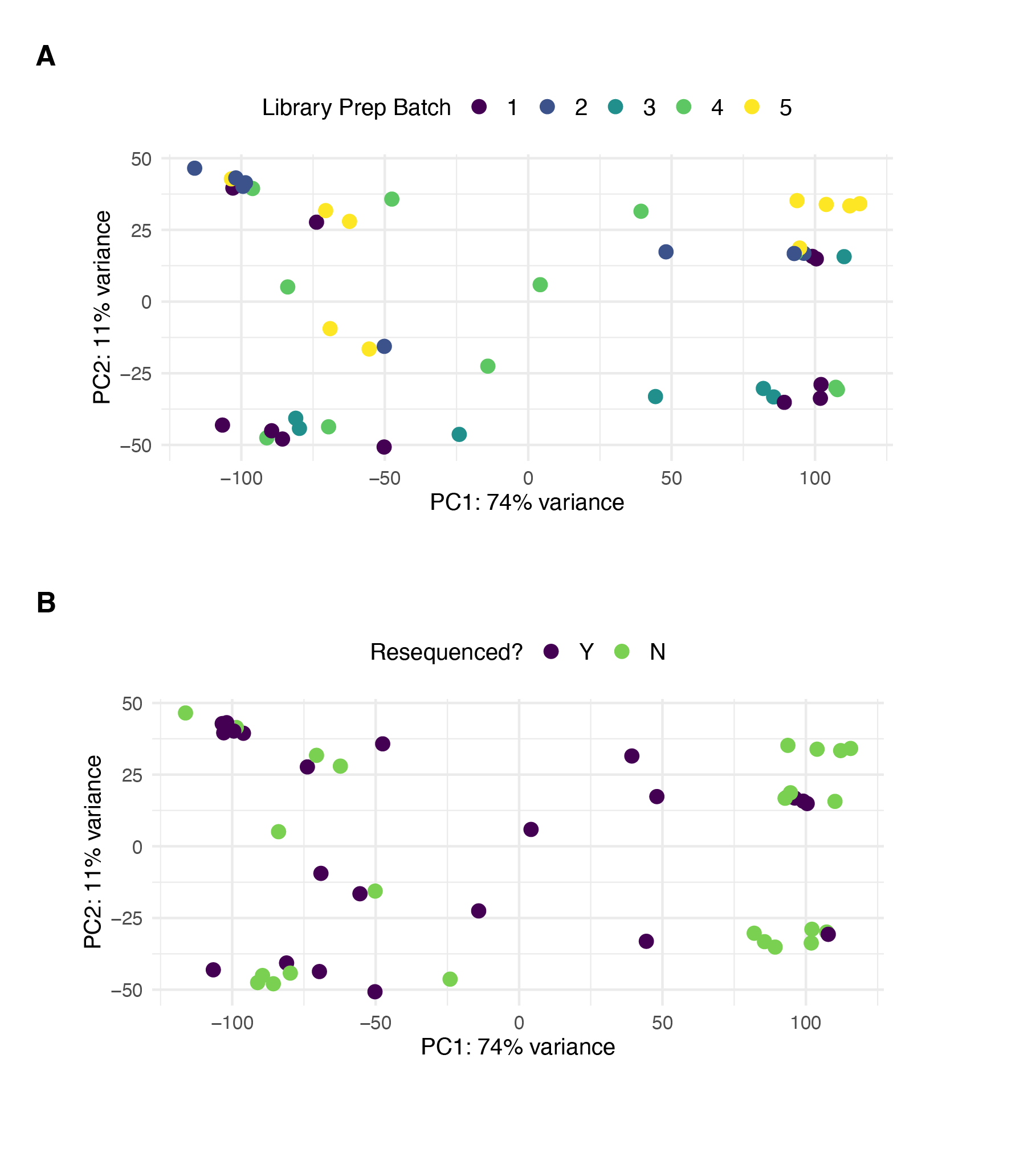

Supplement: S1 Fig — Principal component analysis (PCA) of variance stabilizing transformed gene expression counts for all samples, colored by library preparation batch (A) or sequencing batch (B). Resequencing refers to additional sequencing of some samples to achieve target sequencing depth. (PNG) [file ppat.1012124.s003.png]

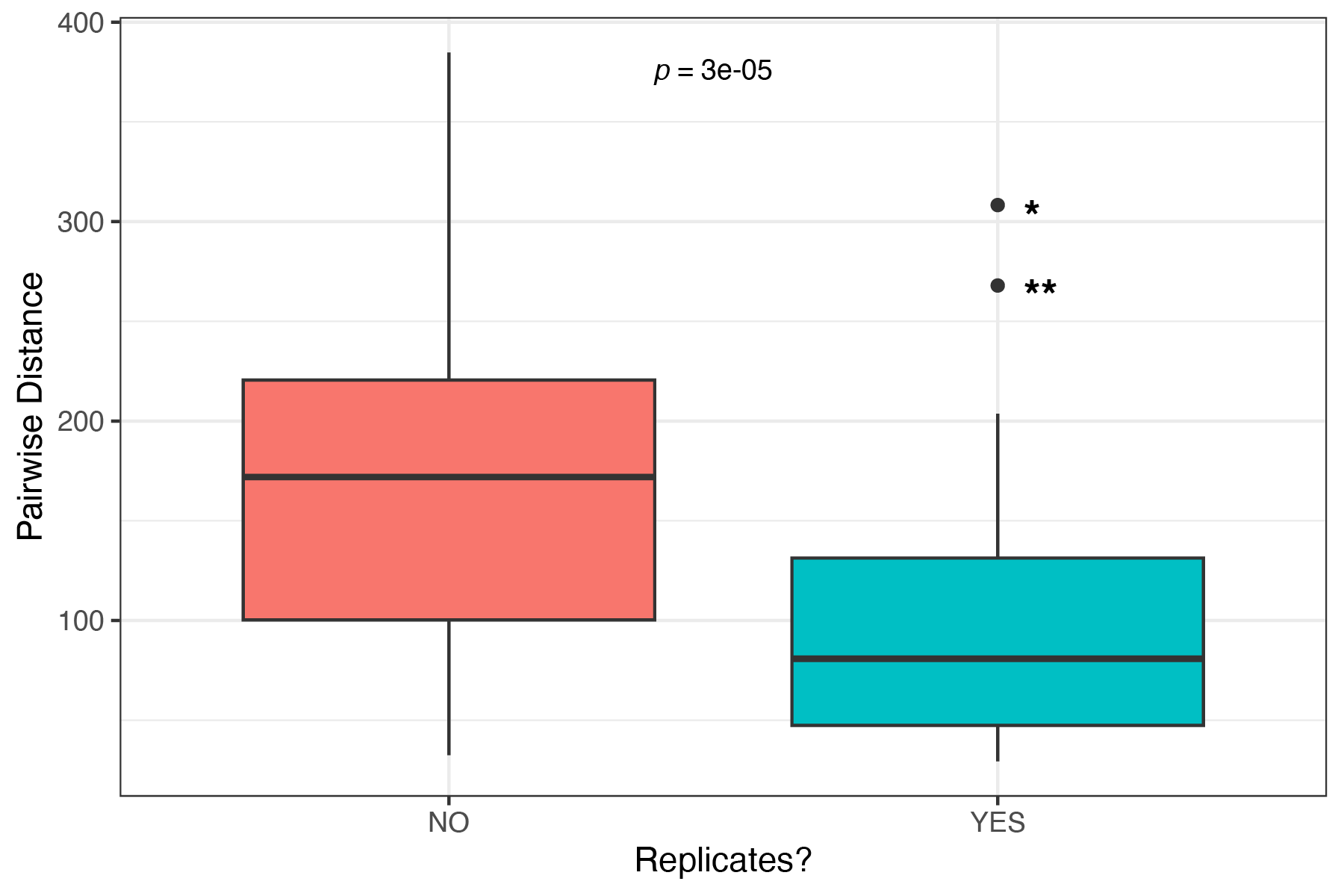

Supplement: S2 Fig — Pairwise distance values calculated from variance stabilizing transformed expression counts between samples that are not biological replicates (NO) and samples that are biological replicates (YES). Biological replicates have significantly shorter distances (Mann Whitney U Test, p = 3e-5) than non biological replicates with two outliers: MT31 evolved planktonic (*) and MT72 ancestral biofilm (**) samples show discordance between their biological replicates which we took into consideration in the analysis of our differential expression results. (PNG) [file ppat.1012124.s004.png]

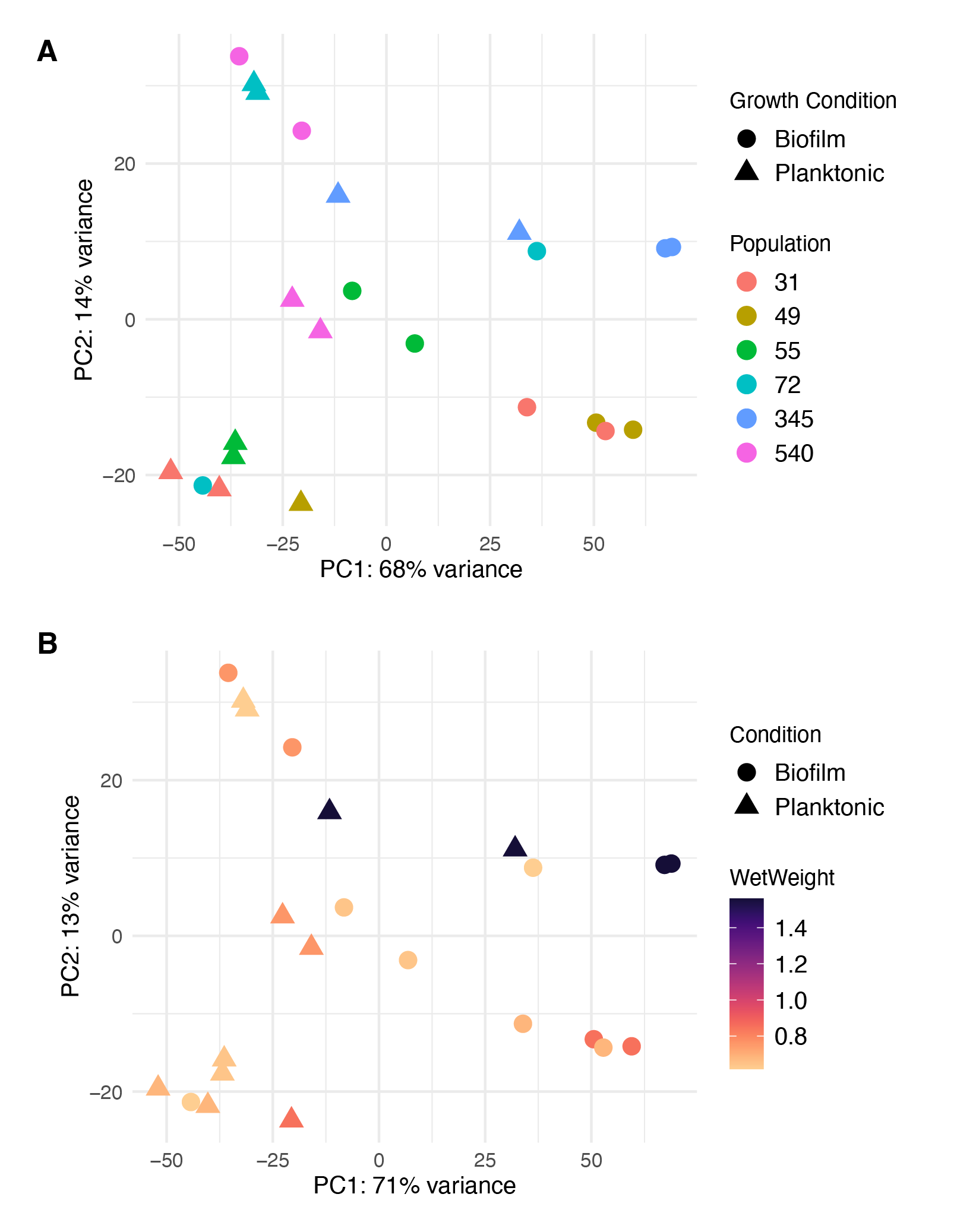

Supplement: S3 Fig — A) Principal component analysis (PCA) of variance stabilizing transformed gene expression counts from ancestral populations. Each point represents the total gene expression of a single sample, where the shape indicates the growth condition of the sample, and the color indicates the population. B) PCA (same as in A) colored this time by biofilm wet weight. There is no clear correlation–either among biofilm samples or planktonic samples–between gene expression patterns and ancestral biofilm phenotype (as measured by wet weight). (PNG) [file ppat.1012124.s005.png]

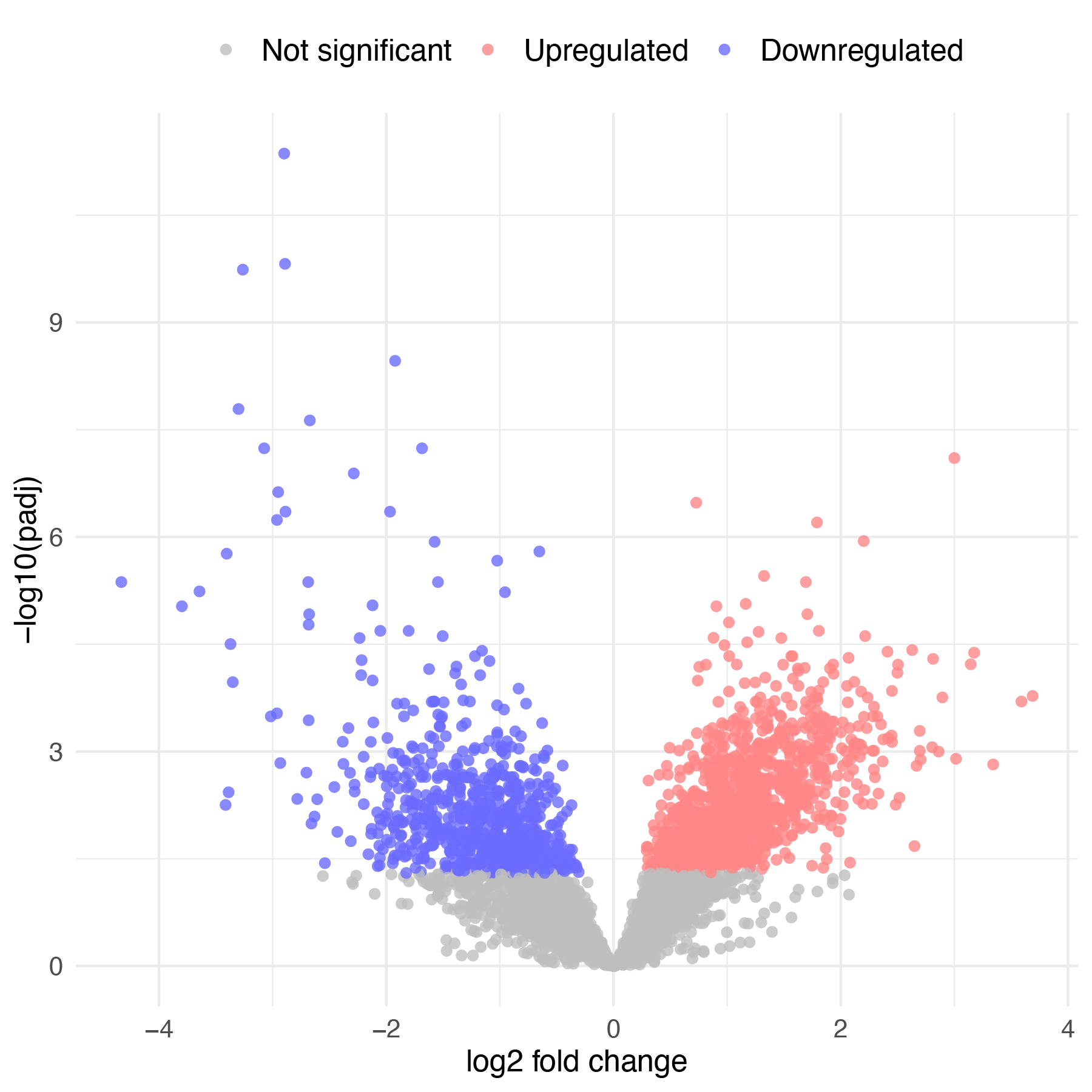

Supplement: S4 Fig — A zoomed in version of Fig 3C. (PNG) [file ppat.1012124.s006.png]

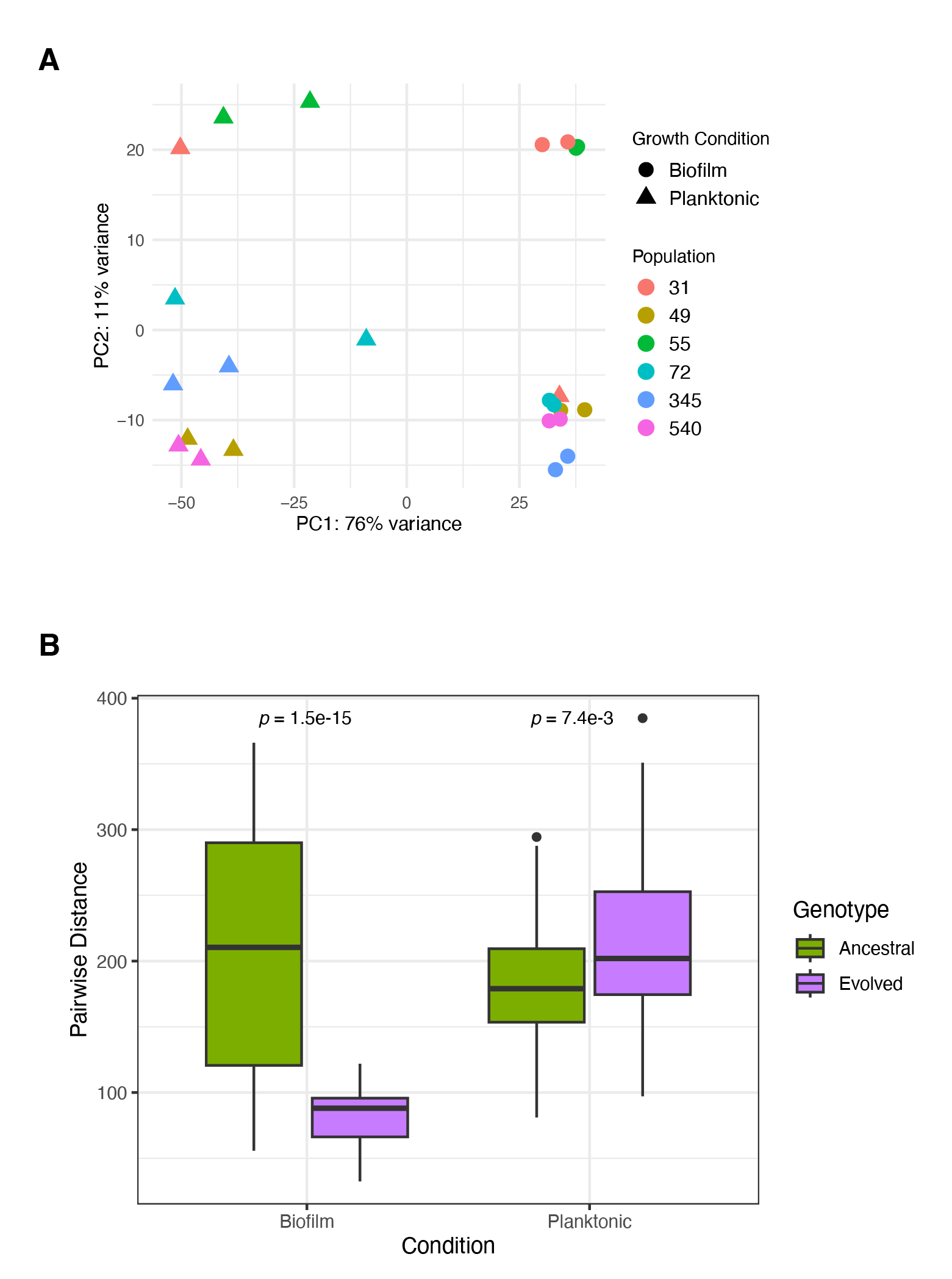

Supplement: S5 Fig — A) Principal component analysis (PCA) of variance stabilizing transformed gene expression counts from evolved populations. Each point represents the total gene expression of a single sample, where the shape indicates the growth condition of the sample, and the color indicates the population. B) Pairwise distance values calculated from variance stabilizing transformed expression counts between samples of the same genotype (ancestral or evolved) and the same growth condition (biofilm or planktonic). Evolved biofilm populations have significantly shorter distances between samples (Mann Whitney U Test with Benjamini-Hochberg correction, p = 1.5e-15) than their ancestral counterparts, indicating more similar gene expression profiles after passaging. Conversely, populations grown in planktonic culture have significantly higher inter-sample distance after passaging (Mann Whitney U Test with Benjamini-Hochberg correction, p = 7.4e-3) indicating that diversity of gene expression under planktonic conditions is unaffected by biofilm passaging. Distances between biological replicates have been excluded. (PNG) [file ppat.1012124.s007.png]

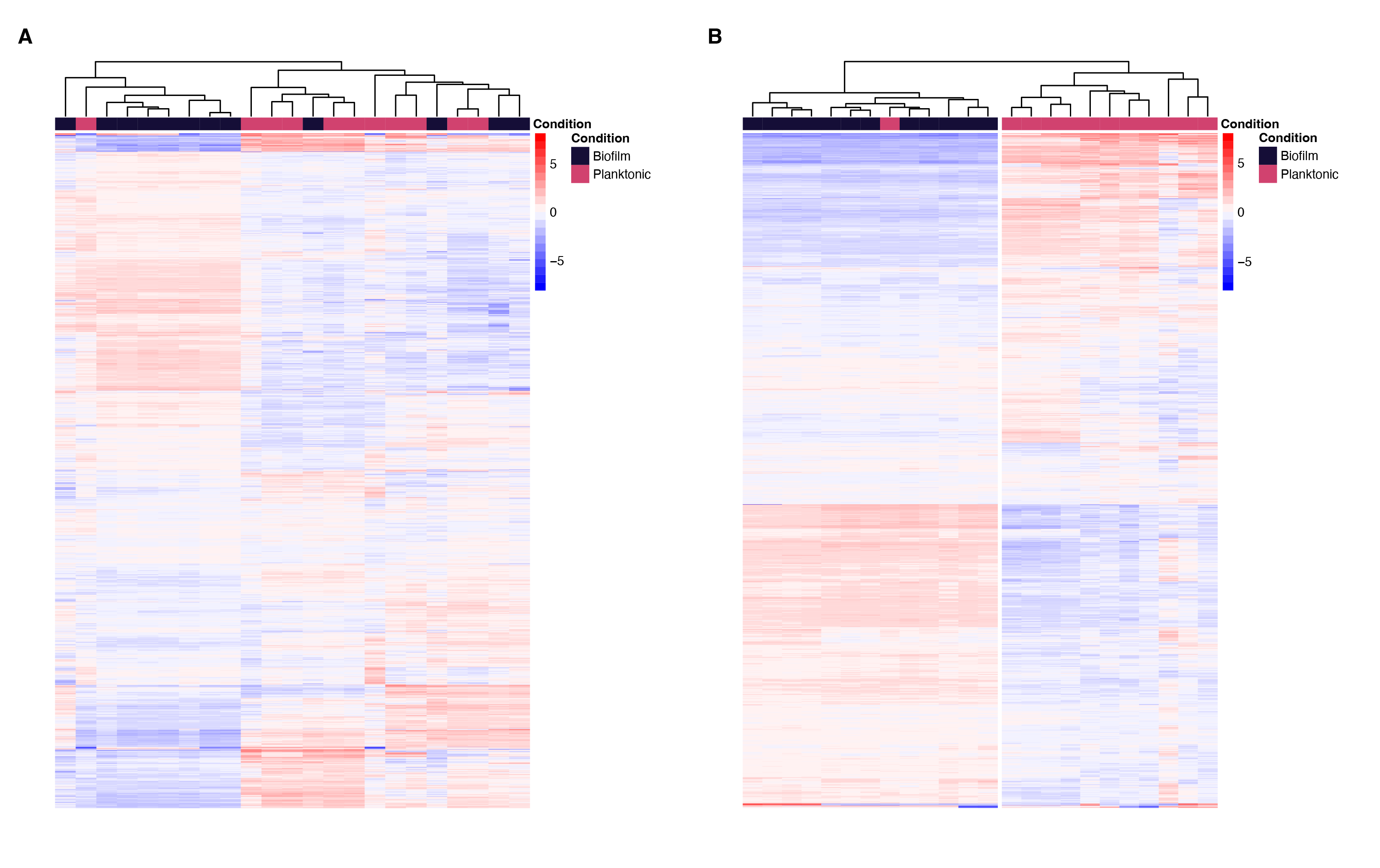

Supplement: S6 Fig — Each column is a single sample from an ancestral (A) or evolved (B) population, grown either as a biofilm or in a planktonic culture. Each row is a gene. Heatmap colored by expression values for each gene which are normalized to the mean across samples. Samples are clustered by Euclidean distance and plotted as a tree at the top of the heatmap. Evolved populations have much more uniform biofilm transcriptomes. (PNG) [file ppat.1012124.s008.png]

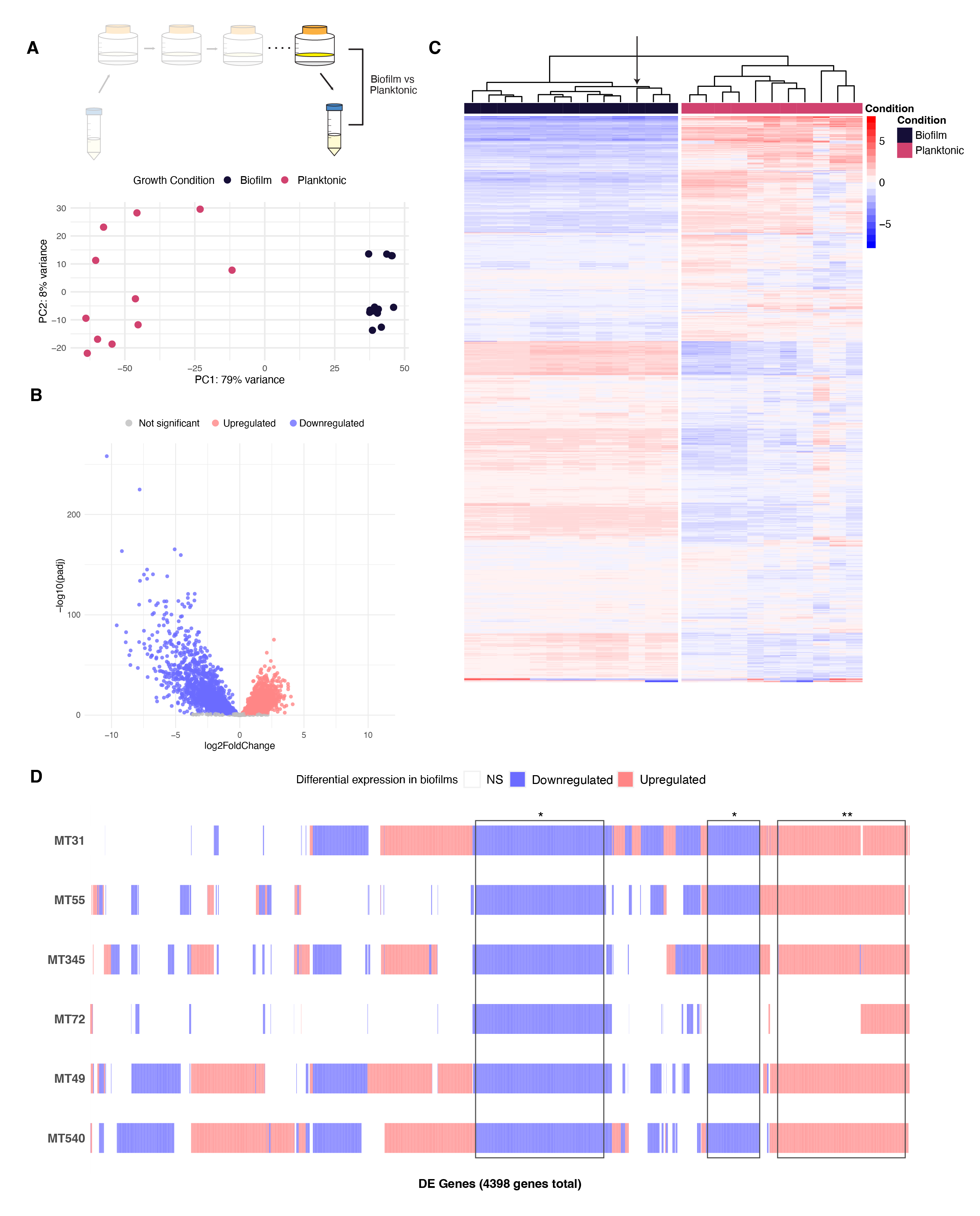

Supplement: S7 Fig — A) Top: Experimental diagram highlighting comparator populations: evolved populations grown as pellicle biofilms are compared to the same populations grown in planktonic cultures. Bottom: Principal component analysis (PCA) of variance stabilizing transformed gene expression for evolved populations grown as biofilms and planktonic cultures. B) Differential expression of DEGs between evolved populations grown as biofilms and as planktonic cultures. Log transformed adjusted p-values plotted against the log2 fold change for each gene. Genes that did not have significant differential expression are shown in grey. C) Heatmap of normalized, variance stabilizing transformed expression counts for DEGs shown in panel B. Each column is a single sample from an evolved population, grown either as a biofilm or in a planktonic culture–the sample identified as an outlier in Fig 4 is highlighted by an arrow. Each row is a DEG. Expression values for each gene are normalized to the mean across samples. Samples are clustered by Euclidean distance and plotted as a tree at the top of the heatmap. D) Matrix of individual DEGs shared across evolved populations. A total of 4373 DEGs are plotted according to if that gene is upregulated (red), downregulated (blue) or not significantly differentially expressed (white) in each population. 24% of downregulated genes are shared by at least 5 populations (*), while 16% of upregulated genes are shared by at least 5 populations (**). (PNG) [file ppat.1012124.s009.png]

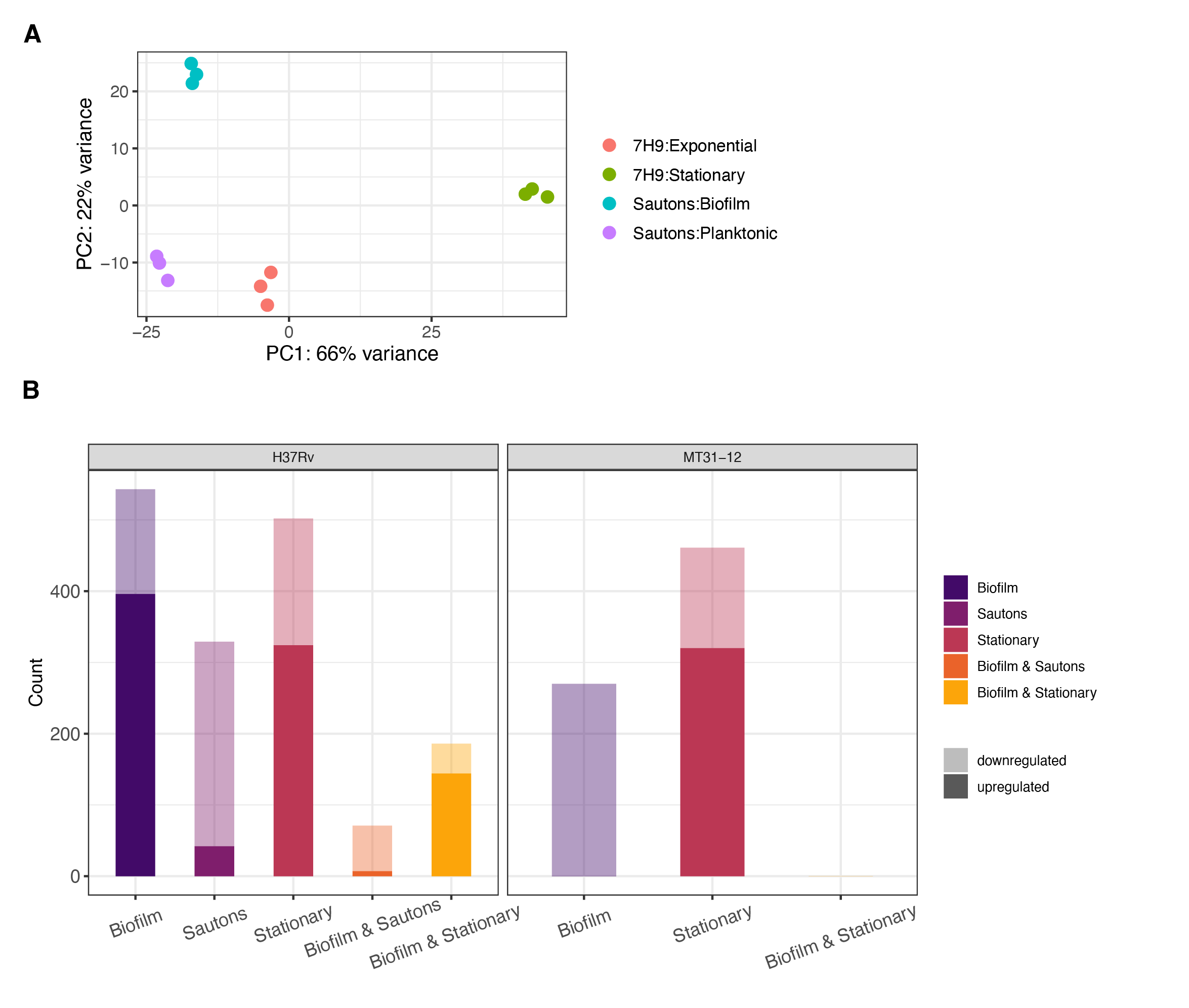

Supplement: S8 Fig — A) Principal component analysis (PCA) of variance stabilizing transformed gene expression for H37Rv grown in different media, and under different growth conditions. Patterns of gene expression associated with four conditions (minimal media, stationary phase, exponential phase, pellicle biofilm) were distinct. B) Counts of highly differentially expressed genes (DEGs, Log2 fold-change >2/<-2) for H37Rv in pellicle biofilms, Sauton’s minimal media, and stationary phase, relative to exponential phase planktonic culture. Counts of DEGs shared among conditions are also shown. C) Highly differentially expressed genes (DEGs, Log2 fold-change >2/<-2) for evolved MT31 in stationary phase vs planktonic, and pellicle biofilms vs planktonic culture. No DEGs were common to both conditions. (PNG) [file ppat.1012124.s010.png]

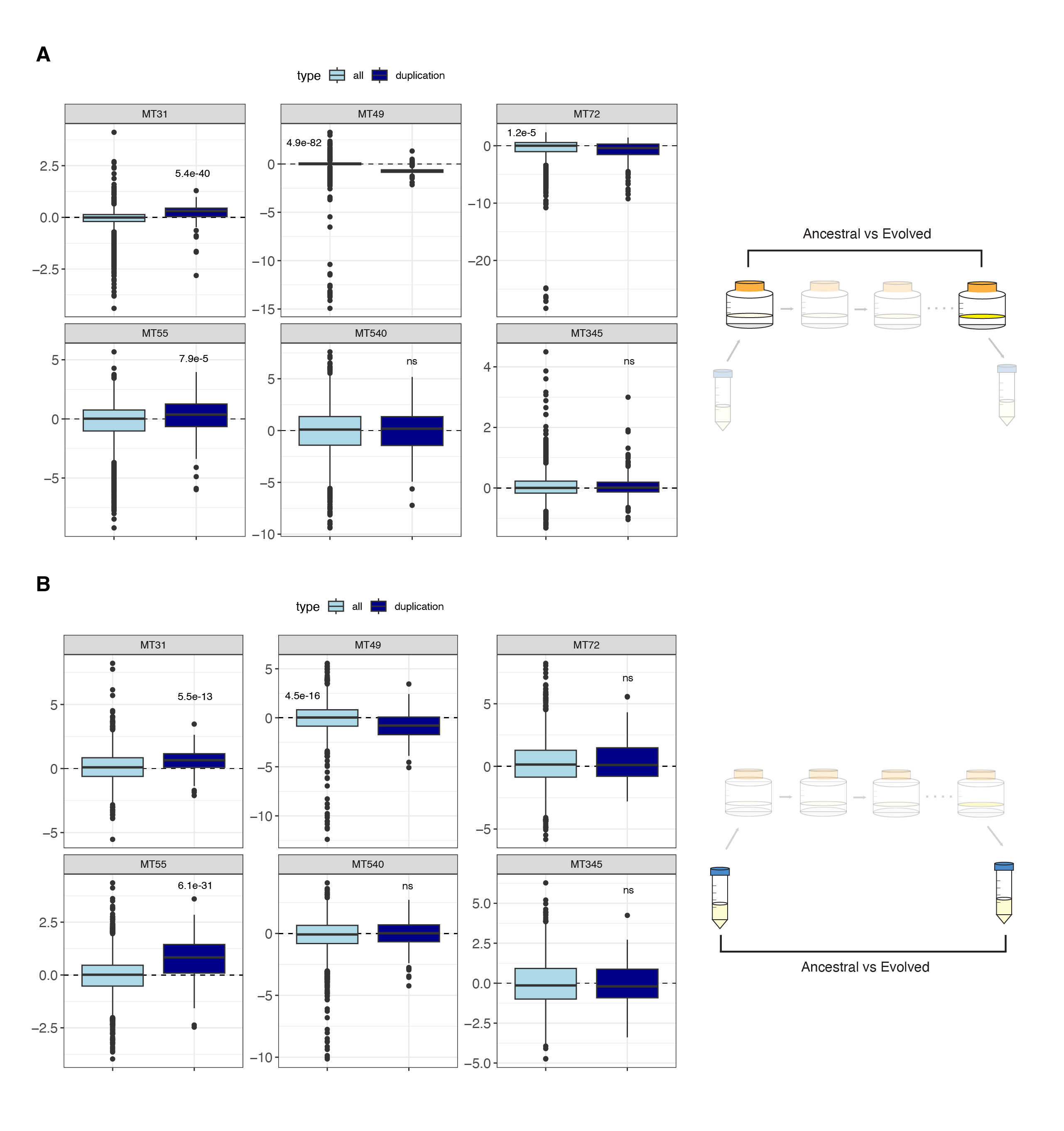

Supplement: S9 Fig — A) Log-2 fold changes (L2FC) for all genes in each biofilm population, separated by their presence in one or both duplications acquired by MT31 and MT55. Mann Whitney U test with Benjamini-Hochberg correction shows significantly higher L2FC values for duplicated genes in MT31 and MT55 (same data shown in Fig 6C), and significantly lower L2FC values for duplicated genes in MT49 and MT72. Comparison is between evolved populations grown as a biofilm and ancestral populations grown as biofilms. B) Same as A, but comparing pellicle evolved populations grown as planktonic cultures, to ancestral populations grown as planktonic cultures. Mann Whitney U test with Benjamini-Hochberg correction shows significantly higher L2FC values for duplicated genes in MT31 and MT55 and significantly lower L2FC values for duplicated genes in MT49. (PNG) [file ppat.1012124.s011.png]

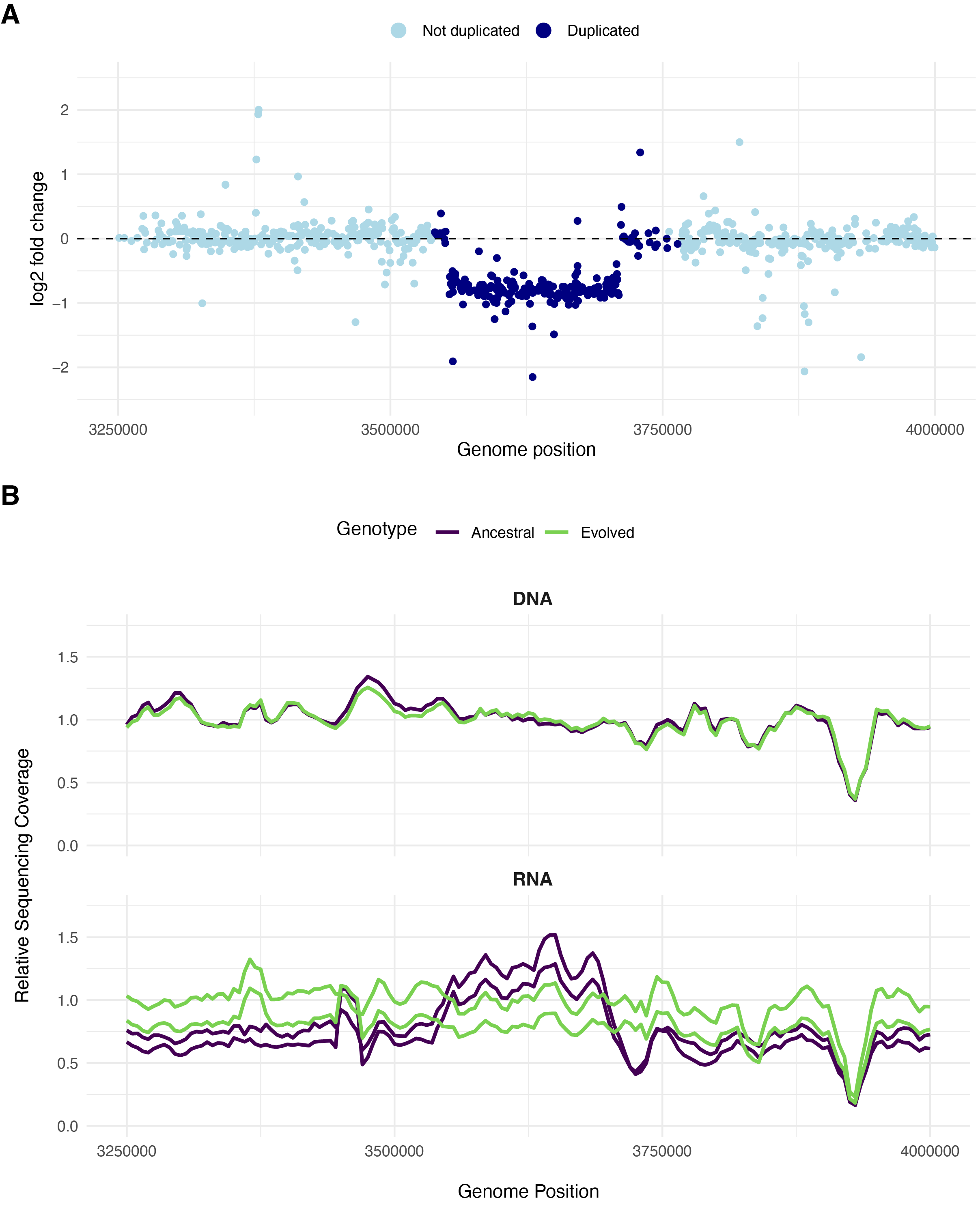

Supplement: S10 Fig — A) log2 fold change (L2FC) values between evolved and ancestral biofilm populations of MT49. Shown is each gene within the region surrounding the duplication which arose in MT31 and MT55. Points are colored according to whether they lie inside of the duplicated region. B) Top: Relative coverage of DNA sequencing for the ancestral (passage 0) and evolved (passage 12) populations of MT49. Bottom: Relative coverage of RNA sequencing for ancestral and evolved populations of MT49. Two lines per genotype indicate two biological replicates. (PNG) [file ppat.1012124.s012.png]

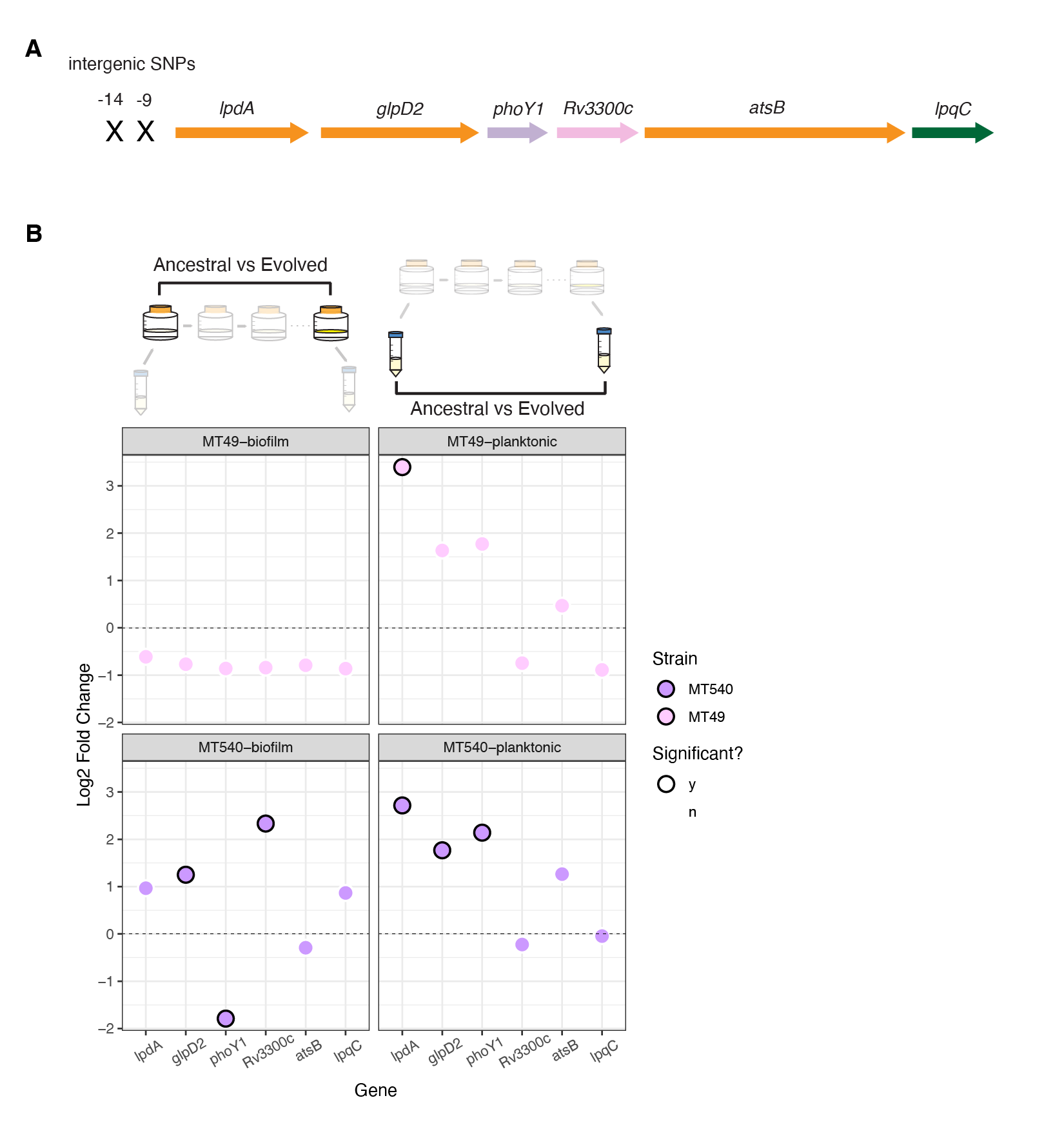

Supplement: S11 Fig — A) Schematic of lpdA operon relative to the two intergenic SNPs acquired by MT49 and MT540 over the course of passaging. Genes are colored by functional annotation from Mycobrowser: orange–Intermediary metabolism and respiration, purple–regulatory proteins, pink–conserved hypotheticals, green—cell wall and cell wall processes. B) Log-2 fold change (L2FC) in expression comparing evolved to ancestral populations, for genes downstream of a convergent intergenic mutation in two of our populations (MT49 and MT540). Points circled in black indicate significant L2FCs in expression between evolved and ancestral populations. ‘Biofilm’ refers to evolved populations grown as a biofilm, compared to ancestral populations grown as a biofilm, and the same is true for ‘planktonic’ as shown in diagrams at the top of the panel. (PNG) [file ppat.1012124.s013.png]

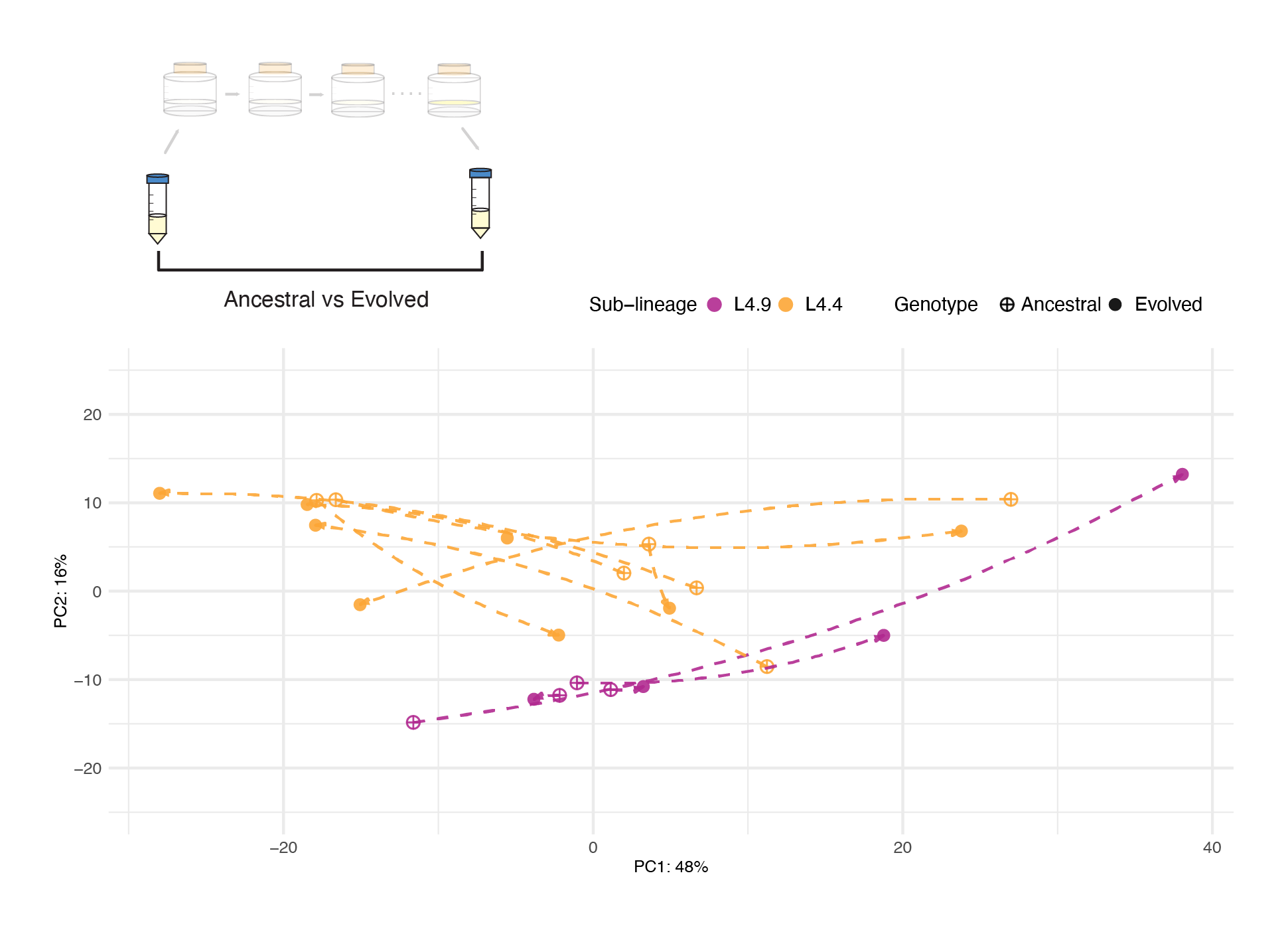

Supplement: S12 Fig — Arrows are drawn between corresponding ancestral and evolved populations, indicating the trajectory of evolution across passaging. Points are colored by sub-lineage of the ancestral population. Note that we do not know the exact evolutionary trajectory of these populations, these lines are merely a graphical representation of that trajectory. (PNG) [file ppat.1012124.s014.png]
